# Vpma phase variation is important for survival and persistence of *Mycoplasma agalactiae* in the immunocompetent host

**DOI:** 10.1371/journal.ppat.1006656

**Published:** 2017-09-28

**Authors:** Rohini Chopra-Dewasthaly, Joachim Spergser, Martina Zimmermann, Christine Citti, Wolfgang Jechlinger, Renate Rosengarten

**Affiliations:** 1 Institute of Bacteriology, Mycology and Hygiene, Department of Pathobiology, University of Veterinary Medicine Vienna, Veterinärplatz 1, Vienna, Austria; 2 UMR1225, INRA, ENVT, Ecole Nationale Vétérinaire, 23 Chemin des Capelles, Toulouse, France; Portland VA Medical Center, Oregon Health and Science University, UNITED STATES

## Abstract

Despite very small genomes, mycoplasmas retain large multigene families encoding variable antigens whose exact role in pathogenesis needs to be proven. To understand their *in vivo* significance, we used *Mycoplasma agalactiae* as a model exhibiting high-frequency variations of a family of immunodominant Vpma lipoproteins *via* Xer1-mediated site-specific recombinations. Phase-Locked Mutants (PLMs) expressing single stable Vpma products served as first breakthrough tools in mycoplasmology to study the role of such sophisticated antigenic variation systems. Comparing the general clinical features of sheep infected with a mixture of phase-invariable PLMs (PLMU and PLMY) and the wild type strain, it was earlier concluded that Vpma phase variation is not necessary for infection. Conversely, the current study demonstrates the *in vivo* indispensability of Vpma switching as inferred from the Vpma phenotypic and genotypic analyses of reisolates obtained during sheep infection and necropsy. PLMY and PLMU stably expressing VpmaY and VpmaU, respectively, for numerous *in vitro* generations, switched to new Vpma phenotypes inside the sheep. Molecular genetic analysis of selected ‘switchover’ clones confirmed *xer1* disruption and revealed complex new rearrangements like chimeras, deletions and duplications in the *vpma* loci that were previously unknown in type strain PG2. Another novel finding is the differential infection potential of Vpma variants, as local infection sites demonstrated an almost complete dominance of PLMY over PLMU especially during early stages of both conjunctival and intramammary co-challenge infections, indicating a comparatively better *in vivo* fitness of VpmaY expressors. The data suggest that Vpma antigenic variation is imperative for survival and persistence inside the immunocompetent host, and although Xer1 is necessary for causing Vpma variation *in vitro*, it is not a virulence factor because alternative Xer1-independent mechanisms operate *in vivo*, likely under the selection pressure of the host-induced immune response. This singular study highlights exciting new aspects of mycoplasma antigenic variation systems, including the regulation of expression by host factors.

## Author summary

Though implicated to play important roles in mycoplasma pathogenicity, the biological significance of large multigene families causing phase variation of immunodominant surface antigens has never been directly proven. Using *M*. *agalactiae* and its Xer1-mediated high-frequency variation system of Vpma surface lipoproteins as a model, we investigated the *in vivo* significance of this variable system by comparing the infection characteristics of two major expression variants, namely VpmaY and VpmaU. ‘Phase-Locked Mutants’ (PLMs) of these expression variants (PLMU and PLMY), served as ideal tools as they steadily express a single Vpma product without further switching. Interestingly, the PLMs altered their Vpma profiles during conjunctival and intramammary co-challenge experiments in sheep despite *xer1* disruption using novel complex switches involving *vpma* chimeras, duplications and deletions. This illustrates that although the Xer1 recombinase is not a virulence factor for *M*. *agalactiae*, Vpma phase variation is important for its survival and persistence in the immunocompetent host. Furthermore, PLMU was outcompeted by PLMY, almost completely at local infection sites during the early stages of infection, indicating a better *in vivo* fitness and survival of VpmaY expressors. This demonstrated the differential infection potential of mycoplasma phase-variable lipoproteins using PLMs in infection studies of the natural host.

## Introduction

Mycoplasmas are not only the smallest but also belong to the most successful bacterial pathogens that cause persistent and often difficult-to eradicate infections in humans and animals [1]. Although a number of mycoplasma diseases have a huge socio-economic significance, proper control strategies are missing, mainly due to lack of knowledge about their pathogenicity mechanisms. They lack typical pathogenicity factors found in other bacteria, and although genomes of many important mycoplasma pathogens have been sequenced, questions pertaining to their virulence and survival remain [1–3]. Having evolved 2.5 billion years ago from a Gram-positive ancestor, mycoplasma evolution was marked by severe genomic reduction, so that contemporary mycoplasmas have lost several metabolic pathways in addition to the genes dedicated to cell wall synthesis. Hence, most mycoplasma species display small genomes while retaining the functions necessary for survival in their respective hosts on which they depend for most of their nutrition [4]. However, many of them devote a sizeable part of their genomes to large multigene families encoding phase- and/or size-variable surface antigens [1, 5]. Although this highlights the biological significance of these multigene-encoded variable antigens in these minimalist organisms, the exact *in vivo* functions are rarely understood in the context of disease progression [6].

Although reversible high-frequency surface variations of bacteria are commonly believed to play a major role in host immune evasion [7, 8], experimental proof of such a role remains circumstantial for mycoplasmas [6]. To our knowledge there is only one *in vivo* study on *M*. *pulmonis* [9], and a couple of *in vitro* or indirect experiments that support the hypothesis that antigenic variation is critical for long-term survival of mycoplasmas, but they do not establish a causal link [5, 6]. In a recent i*n vivo* analysis, Pflaum *et al* [10] have demonstrated similar phase variation events in *M*. *gallisepticum* to be nonstochastic and independent of host adaptive immunity. For most mycoplasma pathogens, the precise role of such multigene-encoded surface lipoproteins and their antigenic switches is yet to be understood, although for *M*. *pulmonis* the Vsa size variations are also shown to modulate adhesion, biofilm formation and protection against complement and phagocytosis [11]. Such studies are hindered due to the cumulative effect of many factors, such as the very high frequency of switching involving large gene families encoding several different clonal variants that are difficult to isolate or distinguish from each other, lack of appropriate animal models, and despite significant advances the continued recalcitrance of mycoplasmas to targeted gene disruptions [12]. Overcoming these challenges, we had earlier constructed *M*. *agalactiae* Phase-Locked Mutants (PLMs) by disrupting the *xer1* gene that encodes the site-specific recombinase causing *vpma* gene inversions responsible for high-frequency switching of the Vpma surface lipoproteins [13, 14]. These PLMs served as breakthrough tools for evaluating the role of such multigene-encoded phase-variable mycoplasma proteins in pathogenesis, as they exhibited stable expression of one defined protein (the ‘locked’ Vpma phenotype) for several *in vitro* generations without further switching to alternate Vpma phenotypes [13]. Besides, *M*. *agalactiae* serves as an excellent model for studying mycoplasma pathogenesis because, unlike many other mycoplasma pathogens, (i) it grows well in the lab, (ii) the pathology associated with disease has been well-documented, (iii) many molecular tools, genomic and proteomic data are available, (iv) different sheep infection models are well-established, and most importantly, (v) it is phylogenetically very close to *M*. *bovis* [15], a worldwide serious pathogen that causes huge economic losses and induces similar clinical signs of mastitis, arthritis and pneumoniae in cattle [3, 16]. Until 1976, *M*. *agalactiae* and *M*. *bovis* were regarded as one species [17], and their genomes show synteny and inversions with many homologous genetic loci [18]. Especially striking is the similarity between the *M*. *bovis* phase-variable Vsp system and the *M*. *agalactiae* Vpma system, as both multigene families have the same conserved 5’ UTR and signal peptides, they contain the same lipoprotein cleavage motif (AAKC), bear repeated sequences, encode similar short cytadherence epitopes, and both undergo site-specific recombination for switching in expression [13, 19, 20]. Hence, Vpmas and Vsps might play similar roles during infection and disease progression, and any leads in our understanding of the role of Vpmas and their phase variation in pathogenesis could be potentially extrapolated to the Vsp system of *M*. *bovis*, and in general to all related mycoplasma phase-variable antigen systems.

Although implicated in host immune evasion, the biological significance of Vpma phase variation is yet to be proven. In a previous study we tried to address this by testing the wild-type *M*. *agalactiae* strain in comparison with the *xer1*-disrupted phase-invariable PLMs in two separate sheep infection models. A mixture of PLMY and PLMU, constitutively expressing VpmaY and VpmaU respectively, and a clonal population of the phase-variable type strain PG2 expressing all six Vpmas were compared for their infection traits in intramammary and conjunctival sheep infections for 28 and 20 days post infection (p.i.), respectively. The study was largely based on clinical data and quantitative mycoplasma loads at various host sites without analyzing their Vpma profiles. The data had demonstrated that although Xer1 recombinase is not a virulence factor of *M*. *agalactiae*, Vpma phase variation might critically influence its persistence during natural infections as suggested by the better dissemination and systemic responses of the phase-variable PG2 infection group [21].

The initial goal of the present study was to check if there were any differences in the colonization potential, *in vivo* fitness, tissue tropism and Vpma-specific humoral responses of Vpma proteins. For this, two major Vpma expression variants of type strain PG2, namely VpmaY and VpmaU, were selected for qualitative analysis of mycoplasma reisolates obtained after PLMY and PLMU co-challenge experiments in the natural sheep host, carried out consecutively *via* the conjunctival and intramammary infection routes, respectively. PLMY showed a marked predominance over PLMU in both infection models implicating a differential pathogenicity potential of Vpma variants. Furthermore, Vpma phenotypic and genotypic analysis of selected reisolates yielded very interesting results, most importantly in two aspects: (i) PLMs which were stable for several generations *in vitro* had ‘switched’ vpmas *in vivo*, and were now expressing new Vpma phenotypes *via* novel Xer1-independent mechanisms as *xer1* was still disrupted, and yet (ii) these clones showed several complex gene rearrangements, including chimeras, deletions and duplications in the *vpma* locus that were never before observed in the PG2 type strain or any clones or PLMs derived from it. Overall, the results highlight the significance of Vpma phase variation for the pathogen’s survival in the immunocompetent host, so much so that although Xer1 is an essential factor responsible for Vpma switching *in vitro*, alternative molecular mechanisms operate in its absence *in vivo*, likely under the selection pressure of the host’s immune response. These are indeed interesting new features of surface antigenic variation systems in pathogenic mycoplasmas.

## Results

### Differential infection potential of Vpma variants in the natural host

#### PLMY and PLMU do not show any competitive growth deficits during *in vitro* mixed culture growth

**Fig 1:**
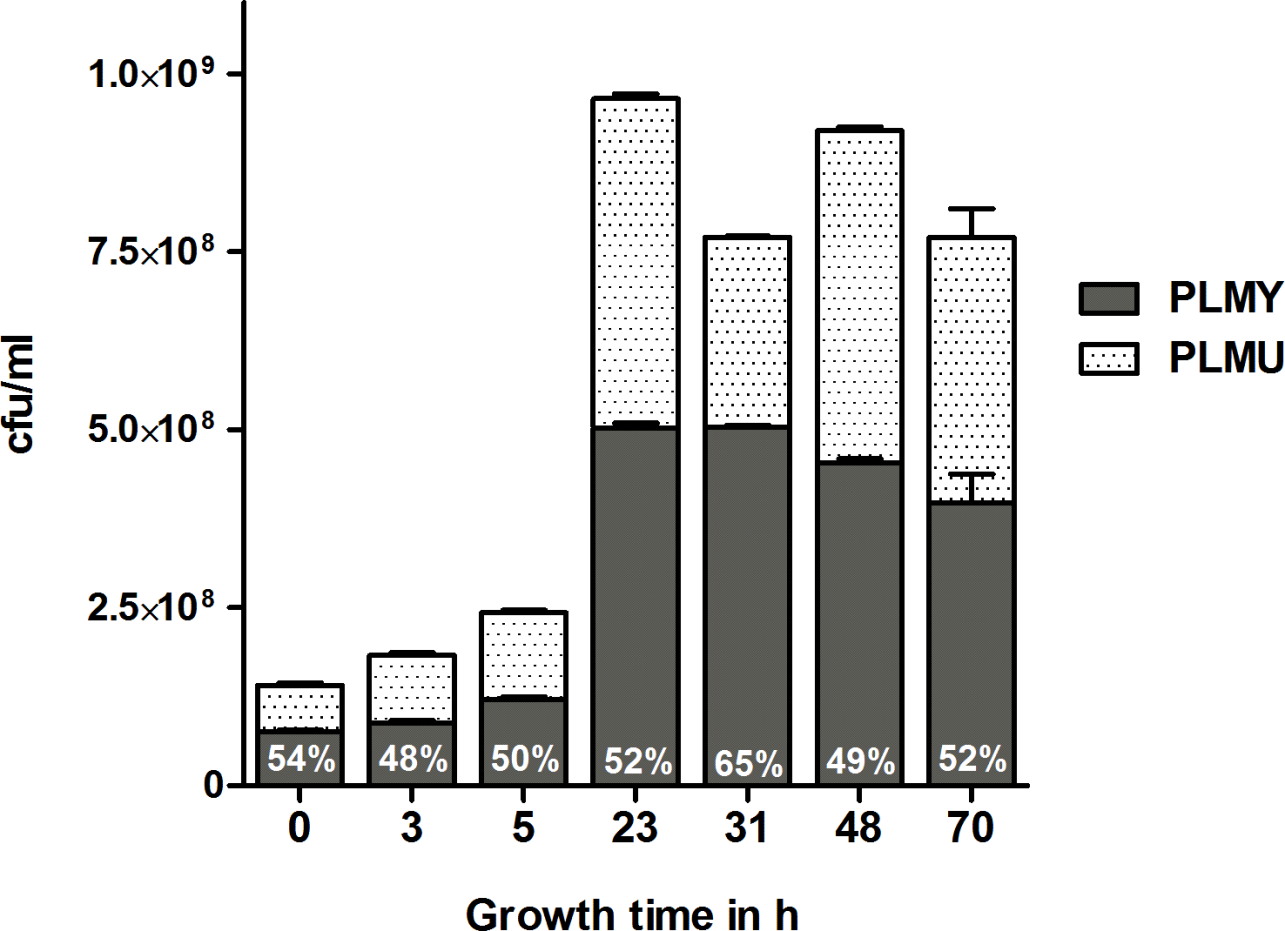
*In vitro* growth profiles of PLMU and PLMY in mixed culture. Samples removed at specific time points were checked for their Vpma expression using colony immunoblotting with Vpma-specific hyperimmune antisera.

To rule out any competitive growth deficits, the PLMU and PLMY inoculum mixture was subjected to an *in vitro* mixed culture growth assay for 70 h where samples were removed at different time points to check the ratios of PLMY and PLMU in the culture *via* colony immunoblotting using VpmaY and VpmaU-specific hyperimmune sera. As shown in Fig 1, no growth advantage or disadvantage was observed for either of the mutants, and all stages of growth demonstrated comparable numbers of PLMU and PLMY in the mixed culture. Absence of all the other four Vpma phenotypes, namely VpmaZ, VpmaX, VpmaW and VpmaV was also confirmed using the respective Vpma specific antisera.

#### PLMY outcompetes PLMU in both intramammary and conjunctival co-challenge experiments

**Fig 2:**
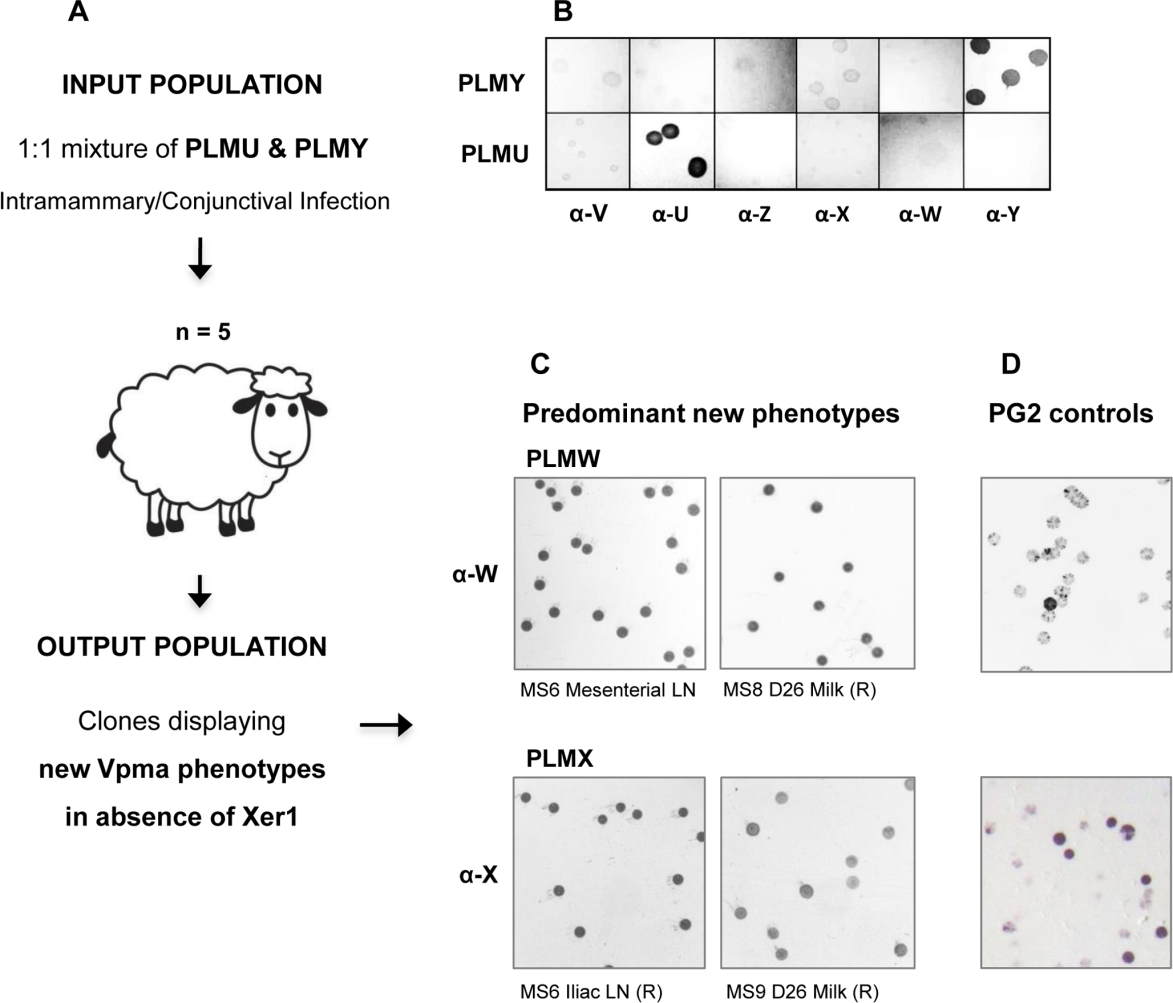
Selection and enrichment of new Vpma ‘switchover’ clones in sheep experimentally infected with PLMU and PLMY. Schematic overview of the sheep infection trials with PLMU and PLMY inoculum (A), which displayed exclusive phase-invariable expression of single VpmaU and VpmaY proteins, respectively, in colony immunoblots with Vpma-specific antibodies for several *in vitro* generations without switching (B). However, inside the immunocompetent host, PLMU and PLMY ‘switched’ to express all the other four alternative Vpma phenotypes, leading to the predominant selection and enrichment of PLMW and PLMX clones, stably expressing VpmaW and VpmaX proteins respectively (C). This predominance was especially noteworthy in some tissues where all the mycoplasma reisolates expressed single VpmaW (for eg. MS 6 / mesenterial LN) or VpmaX (for eg. MS 6 / right iliac LN) proteins, in contrast to the *in vitro* grown phase-variable PG2 population that showed relatively few VpmaW and VpmaX expressors, with most colonies displaying negative or sectored immunostaining patterns corresponding to high-frequency Vpma-switching (D). Colonies of *M*. *agalactiae* isolates were immunostained with the six different Vpma-specific antibodies α-U, α-V, α-W, α-X, α-Y and α-Z. R, right.

Absence of PLMU and almost complete dominance of PLMY was observed in all PLM-challenged sheep at local infection sites during the early stages of infection. For instance, when tested in colony immunoblots with VpmaY- and VpmaU-specific antibodies [13], milk from all five intramammarily-infected sheep showed the predominant presence of PLMY and absence of PLMU right from Day 1 p.i. until Day 8 p.i. as shown in Table 1. At Day 12 p.i., two out of five sheep, i.e., MS 7 and MS 8 started showing positive colonies for PLMU, which by Day 15 p.i. became the 100% prevalent clone in the population in both sheep. In sheep MS 7, this complete dominance persisted until the last sampling of Day 26 p.i., whereas in sheep MS 8, PLMU started getting diluted in milk and was absent by the next sampling on Day 22 p.i. However, beginning at Day 12–15 p.i., most mycoplasmas obtained from the milk of MS 6 and MS 9 expressed neither VpmaU nor VpmaY, as also the case with MS 8 at Day 22–26 p.i. (Table 1). As described in the sections ahead, these mycoplasma isolates from PLMU & PLMY-infected sheep had now ‘switched’ expression to new Vpma phenotypes, predominantly to VpmaW and VpmaX even in the absence of Xer1 recombinase (Table 1, Fig 2). Overall, out of a total of 32 milk samples tested from the infected right udder halves, 17 turned out to be predominantly infected with PLMY alone as compared to 5/32 for PLMU (Table 1). At necropsy, MS 7 was the only sheep that exhibited a predominance of PLMU in all its tested samples, whereas the positive organs and lymph nodes (LN) of the other four sheep either showed a predominance of PLMY or an absence of both PLMU and PLMY (Table 2). These results were consistent with the exceptional predominance of PLMU in the milk of MS 7 sheep starting from Day 15 p.i. (Table 1).

**Table 1:** Vpma phenotypes of *M*. *agalactiae* recovered in milk during the course of intramammary infection.

Sheep ID	Days p.i.^1^							Predominant ‘switchover’ phenotypes^2^
	1	5	8	12	15	22	26	
**MS 6**	Y+	Y+	Y+	Y+	Y-^0.2^	Y-	Y-	VpmaW VpmaX
	U-	U-	U-	U-	U-	U-	U-	
**MS 7**	Y+	Y+	Y+	Y+^m^	Y-	Y-	Y-	NA
	U-	U-	U-	U-^0.5^	U+	U+	U+	
**MS 8**	Y+	Y+	Y+	Y-	Y-	Y-	Y-	VpmaW VpmaX
	U-	U-	U-	U+	U+	U-	U-	
**MS 9**	Y+	Y+	Y+	Y-	Y-	Y-	Y-	VpmaX
	U-	U-	U-	U-	U-	U-	U-	
**MS 10**	Y+	Y+	Y+	NC	Y+^50^	No Milk	No Milk	NA
	U-	U-	U-		U-			

^1^ Comparative viable counts of PLMU and PLMY obtained by colony immunoblotting using α-Y and α-U antibodies. Y and U denote clones expressing VpmaY and VpmaU, respectively. Yellow cells correspond to Y expressors, green to U expressors and pink to neither U nor Y expressors.

^m^ majority of positive cfu (0.1–0.5% negative cfu); 0.2, 0.5 and 50 denote the percentage of positive cfu counts; NC: No results due to sample contamination; NA: Not applicable; No Milk: due to agalactia.

^2^ For Day 26 p.i. milk samples, which showed neither VpmaU nor VpmaY expression, new ‘switchover’ Vpma phenotypes were observed using the other four monospecific Vpma antisera (α-W, α-X, α-V and α-Z Abs)

**Table 2:** Predominant Vpma phenotypes of *M*. *agalactiae* reisolates from udders and lymph nodes of sheep inoculated *via* the intramammary route.

Sheep ID	Lymph nodes and udder samples^a^										
	SUPM L	SUPM R	ILI L	ILI R	MES	MED	CSUP L	PAR L	PAR R	Ud L	Ud R
**MS 6**	NR^b^	NR	Y+ 21	Y-	Y-	NR	NR	NR	NR	Y+ 93	Y+ 25
			U-	U-	U-					U-	U-
				**VpmaX**^**d**^	**VpmaW**						
**MS 7**	Y+ 33^**c**^	Y -	NR	NR	NR	NR	Y-	Y-	Y-	Y+ 2	Y-
	U+ 67	U+					U+	U+98	U+	U+ 98	U+ 97
**MS 8**	NR	NR	Y+ 86	Y-	Y+ 83	Y-	NR	NR	NR	Y+ 0.3	Y-
			U+ 13	U-	U-	U-				U+ 2	U+ 24
				**VpmaW**		**VpmaW**					
**MS 9**	NR	NR	NR	NR	NR	NR	NR	NR	NR	NR	Y-
											U-
											**VpmaV**
											**VpmaX**
**MS 10**	NR	NR	NR	NR	NR	NR	NR	NR	NR	Y-	NR
										U-	
										**VpmaV**	

VpmaU and VpmaY phenotyping using α-U and α-Y rabbit antisera, respectively, was performed on mycoplasma reisolates from lymph nodes and udders of sheep necropsied on Day 28 p.i. Yellow cells correspond to predominance of VpmaY expressors, green to U expressors and pink to neither U nor Y.

^a^ Lymph node and Ud (udder) L (left) / R (right)–SUPM: supramammary; ILI: iliac; MES: mesenterial; MED: mediastinal; CSUP: superficial cervical; PAR: parotideal

^b^ NR: No *M*. *agalactiae* isolation after direct plating indicating a very low initial mycoplasma load.

^c^ Percentage of VpmaY or VpmaU expressors; + and–without percentage numbers denote 100 and 0 percent positive cfu, respectively.

^d^ Predominant Vpma phenotypes of ‘switchover’ clones when neither VpmaY nor VpmaU was expressed

The conjunctival infection in lambs also yielded similar results. Even though the general mycoplasma loads were comparatively much lower in this case [21], all the seven tested nasal and eye swabs of Day 1 –Day 9 p.i. positive for mycoplasma reisolation demonstrated a predominance of PLMY (Table 3). The two positive eye swabs of Day 2 p.i., namely from sheep S 9 and S 10, showed that all the 44 and 36 cfu respectively, constituted PLMY, and that PLMU was completely absent. Similar 100% dominance of PLMY was observed for eye swabs of sheep S 7, S 8 and S 10 taken on Day 9 p.i. The single positive nasal swab tested for Day 1 p.i. yielded 59% PLMY clones compared to 22% PLMU (Table 3). At necropsy, 8 out of 10 tested positive LNs showed a higher percentage of PLMY clones compared to PLMU as depicted in Table 4. For instance, PLMU was completely absent (0%) in right mandibular LN of sheep S 6 and S 7, whereas PLMY constituted 91% and 74%, respectively, of the total mycoplasma reisolates. Similarly, lateral retropharyngeal LN from sheep 7 showed 81% PLMY and 17% PLMU, whereas sheep 9 showed 20% PLMY and 1% PLMU in the total mycoplasma load (Table 4).

**Table 3:** Vpma phenotypes of *M*. *agalactiae* recovered from the nasal and eye swabs of conjunctival route infected sheep.

Sheep No. / Day p.i. / Swab ^a^	Percentage Presence		
	PLMY	PLMU	
**S 9 / 1 / NR**	59%		22%
**S 9 / 2 / ER**	100%		0
**S 10 / 2 / ER**	100%		0
**S 7 / 9 / ER**	100%		0
**S 8 / 9 / ER**	100%		0
**S 9 / 9 / ER**	86%		0
**S 10 / 9 / ER**	100%		0

VpmaU and VpmaY phenotyping (using α-U and α-Y rabbit antisera, respectively) of eye and nasal swabs, which were positive for mycoplasma reisolation after direct plating of frozen samples.

^a^ Swab samples: N- nasal swab; E- eye swab; R- right

**Table 4:** Vpma phenotypes of *M*. *agalactiae* reisolates from lymph nodes of sheep inoculated *via* the conjunctival route.

Sheep ID	Lymph nodes ^a^				
	MAN R	PAR R	RPHM L	RPHM R	RPHL R
**S 6**	Y+ 91^b^	Y+ 90	NR	NR	NR
	U-	U-			
**S 7**	Y+ 74	Y+ 68	NR	Y+ 81	Y+ 40
	U-	U+ 4		U+ 17	U+ 49
**S 8**	NR^c^	NR	NR	NR	NR
**S 9**	NR	Y+ 18	Y+ 20	NR	NR
		U+ 80	U+ 1		
**S 10**	Y+ 98	Y+	NR	NR	NR
	U+ 1	U-			

VpmaU and VpmaY phenotyping, using α-U and α-Y rabbit antisera, respectively, was performed on mycoplasma reisolates from lymph nodes of sheep necropsied on Day 20 p.i. Yellow cells correspond to predominant VpmaY expressors and green to U expressors.

^a^ Lymph node L (left) / R(right)–MAN: mandibular; PAR: parotideal; RPHM: medial retropharyngeal; RPHL: lateral retropharyngeal.

^b^ Percentage of VpmaY or VpmaU expressors; + and–without percentage numbers denote 100 and 0 percent positive cfu, respectively.

^c^ NR: No *M*. *agalactiae* isolation after direct plating indicating a very low initial mycoplasma load.

Mycoplasma reisolates obtained from the PG2-infected sheep exhibited a complex mix of all six Vpma phenotypes in the same sample with no particular predominance for any one of these in most cases. Moreover, Xer1-mediated site-specific recombination in the *vpma* locus of wild type PG2 strain caused very high-frequency Vpma switching resulting in highly sectored colony phenotypes that could not be quantified precisely. However, enrichment of VpmaY expressors over VpmaU was clearly visible in few samples where VpmaU was found to be completely absent, for instance in the eye swabs of sheep S 11 on Day 2 and 9 p.i and S 14 on Day 9 and 19 p.i.

The results of both the conjunctival and the intramammary sheep infection experiments demonstrate that mycoplasmas expressing VpmaY have a better fitness *in vivo* than those expressing VpmaU. This growth difference is exclusively an *in vivo* characteristic as PLMU does not show any growth retardation and the *in vitro* growth rates of PLMU and PLMY are comparable (Fig 1). However, the conjunctival infection model did not lead to any clinical signs, and the few samples that were found positive for mycoplasmas, yielded very low cfu counts. Hence, hereafter the study will mainly concentrate on the intramammary infection model.

#### Predominance of PLMY-specific antibodies in sheep

**Fig 3:**
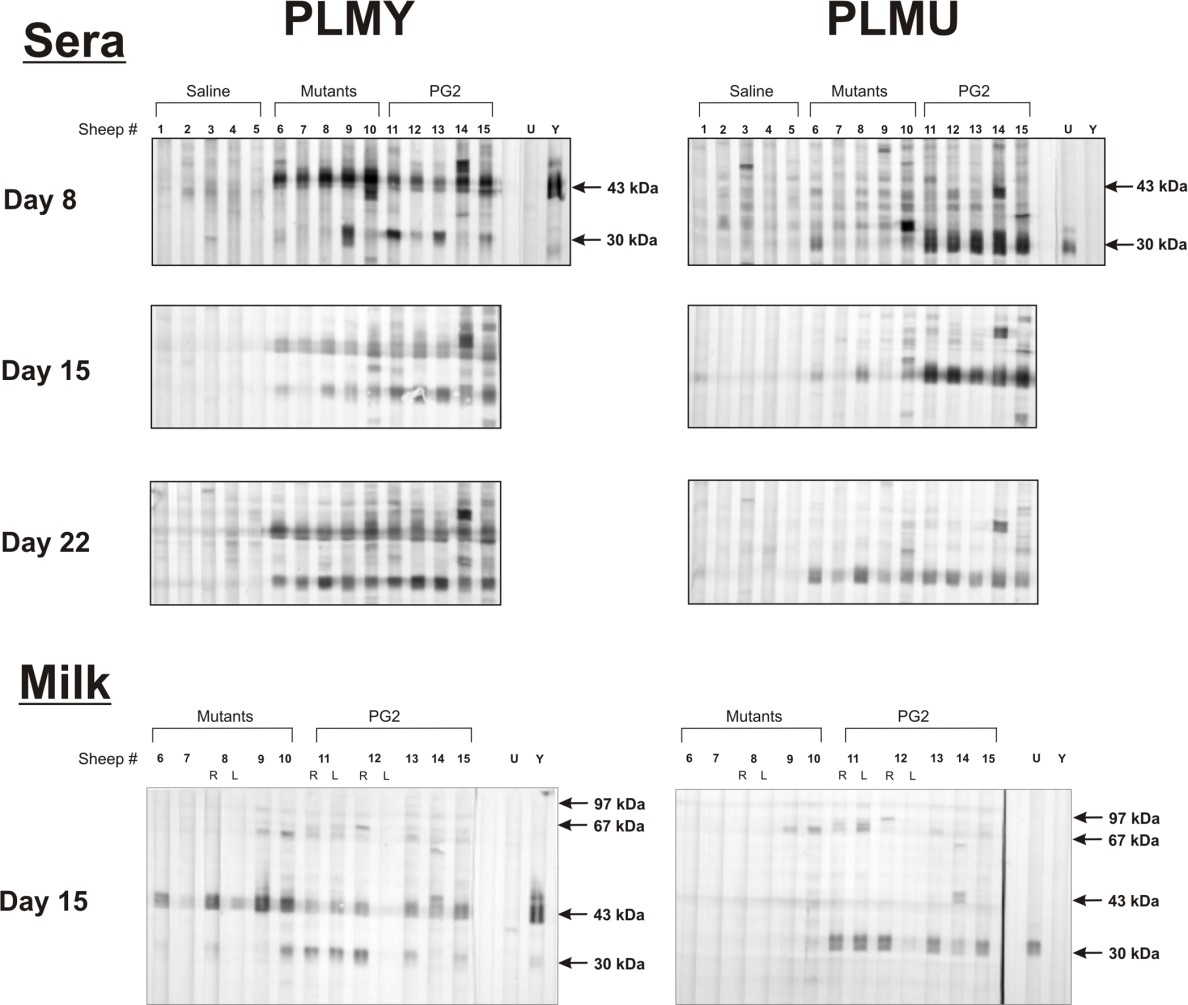
Predominance of PLMY-specific antibodies in sheep sera and milk. Western blot analysis of PLMY and PLMU whole cell proteins using sera and milk from individual sheep inoculated with saline (sheep MS 1–5), the PLMU and PLMY mutant mixture (sheep MS 6–10) or the WT PG2 strain (sheep MS 11–15). Y and U represent controls with monospecific VpmaY- and VpmaU-specific rabbit antisera α-Y and α-U, respectively. L represents milk from left udder halves, whereas R and unmarked samples represent milk from the inoculated right udder halves.

A strong antibody response against PLMY was evident in the sera of sheep infected with a mixture of PLMU and PLMY starting at Day 8 p.i. and lasted until the end of the infection trial (Fig 3). On the contrary, a PLMU-specific response was missing at Day 8 p.i. and became noticeable in some sheep only at later stages of infection, i.e. after Day 15 to Day 22 p.i. This correlates well with the relative predominance of PLMY clones in sheep infected with the PLMs. As evidenced by the specific rabbit antisera controls in the Western blots of Fig 3, PLMY and PLMU induced antibody responses against their corresponding VpmaY and VpmaU proteins respectively. As the primary antibody in ruminant milk is IgG rather than IgA [22], anti-VpmaY/VpmaU IgG antibodies were tested in the milk samples using the same set-up as used for investigating the sheep sera. A predominance of VpmaY-specific antibodies was also observed in the milk samples obtained at Day 15 p.i., whereas a VpmaU-specific immune response was absent in milk at this time (Fig 3). Interestingly, sheep inoculated with *M*. *agalactiae* type strain PG2 could mount a VpmaU-specific antibody response as observed in sera obtained at all tested time points starting from Day 8 p.i., as well as in Day 15 p.i. milk samples (Fig 3). This is in contrast to the sera/milk from PLM-infected sheep that exclusively recognize PLMY during Western blot analysis until Day 15 p.i. (Fig 3).

#### *In vivo* selection and enrichment of VpmaW and VpmaX expressors

**Fig 4:**
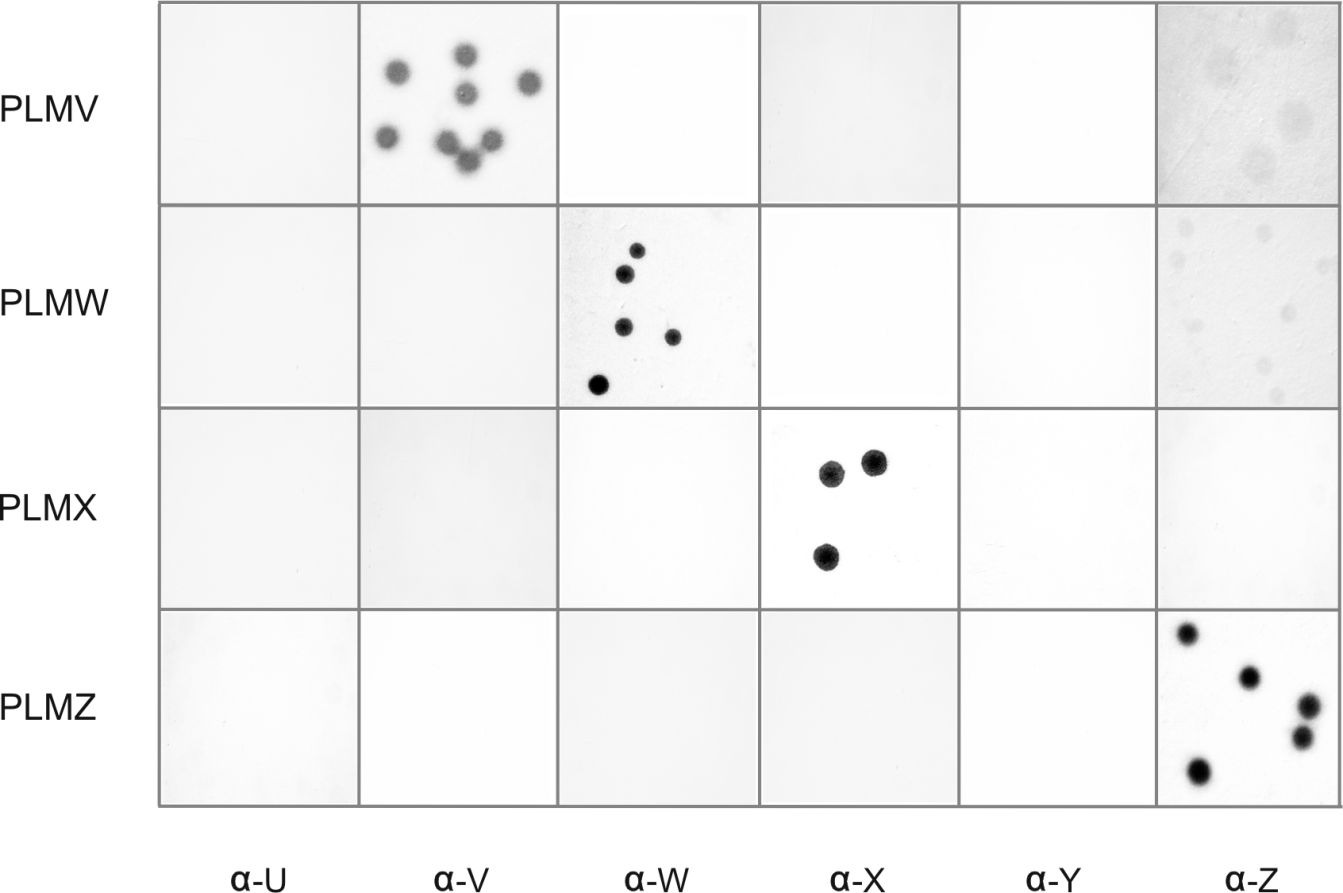
*In vivo* generation of new PLM phenotypes. Colony immunoblots of four different representative PLM ‘switchover’ clones reisolated from sheep experimentally infected with a mixture of PLMU and PLMY. Each of the four PLMs show positive immunostaining only with a single specific pAb and are negative with all the other five Vpma-specific pAbs.

It is known that the Xer1 recombinase controlling Vpma switching functions preferentially in cis, and clonal variants expressing VpmaY, VpmaU and VpmaZ are the most prevalent, whereas VpmaW, VpmaV and VpmaX expressors constitute a minor part of the *in vitro* grown PG2 population [13, 20, 23]. However, a considerable enrichment of these minor phenotypes was observed *in vivo* (Figs 2 and 4) during the sheep intramammary infections with a mixture of PLMY and PLMU, which had been previously shown to be phase-invariable *in vitro* [13]. Compared to the very few VpmaW and VpmaX expressors in the wild type PG2 population *in vitro* (Fig 2D), enrichment of VpmaW and VpmaX variants was most conspicuous in almost all positive samples at necropsy that had stopped the expression of VpmaU and VpmaY. In some tissue samples, VpmaW expressors showed 100% prevalence and all other Vpma clonal variants were completely absent, for instance, in mesenterial LN of MS 6 and mediastinal LN of MS 8 (Fig 2C, Table 2, S1 Fig). A similar 100% prevalence of VpmaX expressors was observed in right iliac LN of MS 6 (Fig 2C), whereas many other samples exhibited a comparatively larger proportion of clones expressing VpmaX (Tables 1 and 2). Although VpmaV and VpmaZ expressors were also isolated from some sheep samples, the proportion and frequency of VpmaW and VpmaX isolations was comparatively much higher.

Additionally, VpmaX and VpmaW were also the most predominant ‘switchover’ phenotypes of mycoplasma milk reisolates that had repressed the expression of both VpmaU and VpmaY by Day 26 p.i. (Table 1) although VpmaZ and VpmaV variants were observed in milk from sheep MS 8 and MS 10, respectively

Although in general the analysis of PG2 reisolates *via* colony immunoblotting was difficult due to the predominant sectorial phenotype, α-W colony immunoblots of some samples showed majority of colonies to be non-sectored and completely positive as compared to the PG2 inoculum control where the proportion of such colonies is ˂10%. This pointed towards an enrichment of the VpmaW phenotype in some of the PG2-infected sheep samples, such as the mesenterial LN (of sheep MS 11 and MS 13), in the right iliac LN (of MS 11, 12 and 13), left iliac LN (MS 14), right udder and right supramammary LN (of MS 15). Similarly, VpmaX predominance was observed in the right and left iliac LNs of MS 11 and MS 14, respectively.

### *In vivo* generation of new Vpma phenotypes in absence of Xer1 site-specific recombinase

Absence of VpmaY and VpmaU phenotypes (corresponding to the PLMY and PLMU input population) in various milk samples obtained at Days 22–26 p.i. (Table 1) was an interesting result, and initially we thought that it could have two main implications: (i) either the PLMs had stopped their Vpma expression altogether, or, (ii) contrary to their so far known *in vitro* stability and phase-invariable phenotypes, they had now switched to express other Vpmas *in vivo*, even in the absence of Xer1 recombinase, the molecular switch that was earlier shown to be necessary for Vpma phase variations [13]. To check this hypothesis, milk and other necropsied tissue samples that showed neither VpmaU nor VpmaY expression (S2 Fig) were tested for Vpma phenotypes other than VpmaY and VpmaU. Results confirmed that the PLMs had indeed changed their Vpma profiles *in vivo* to express one of the other four Vpmas that were initially not expected, namely VpmaZ, VpmaW, VpmaV and VpmaX (Fig 2, Tables 1 and 2, S1 and S3 Figs). For instance, S3 Fig is a representative figure that shows the expression of the latter three Vpmas by such ‘switchover’ clones in the right udder of MS 8. Similarly, S1 Fig shows the ‘switchover’ expression of VpmaW in the mesenterial LN of MS 6. However, when picked and filter cloned, the majority of the ‘switchover’ clones did not undergo further switching like the phase-variable WT strain and behaved as new PLMs with alternate Vpma phenotypes as shown in Fig 4. They seemed to be ‘locked’ for the expression of only one Vpma and gave positive colonies (without sectoring) only with the corresponding Vpma-specific pAb, and were negative with all the other five Vpma-specific pAbs (Fig 4). Hence, PLMY and PLMU, which were stable for several *in vitro* generations encountered selection pressure in the immunocompetent host to switch to different Vpma phenotypes allowing us to select new PLMs, namely PLMW (from mesenterial LN of sheep MS 6), PLMZ (from right parotideal LN of sheep S 7), PLMV (from right udder of sheep MS 10) and PLMX (from right udder of sheep MS 9) (Figs 2 and 4, Tables 1 and 2, S1 and S3 Figs).

**Fig 5:**
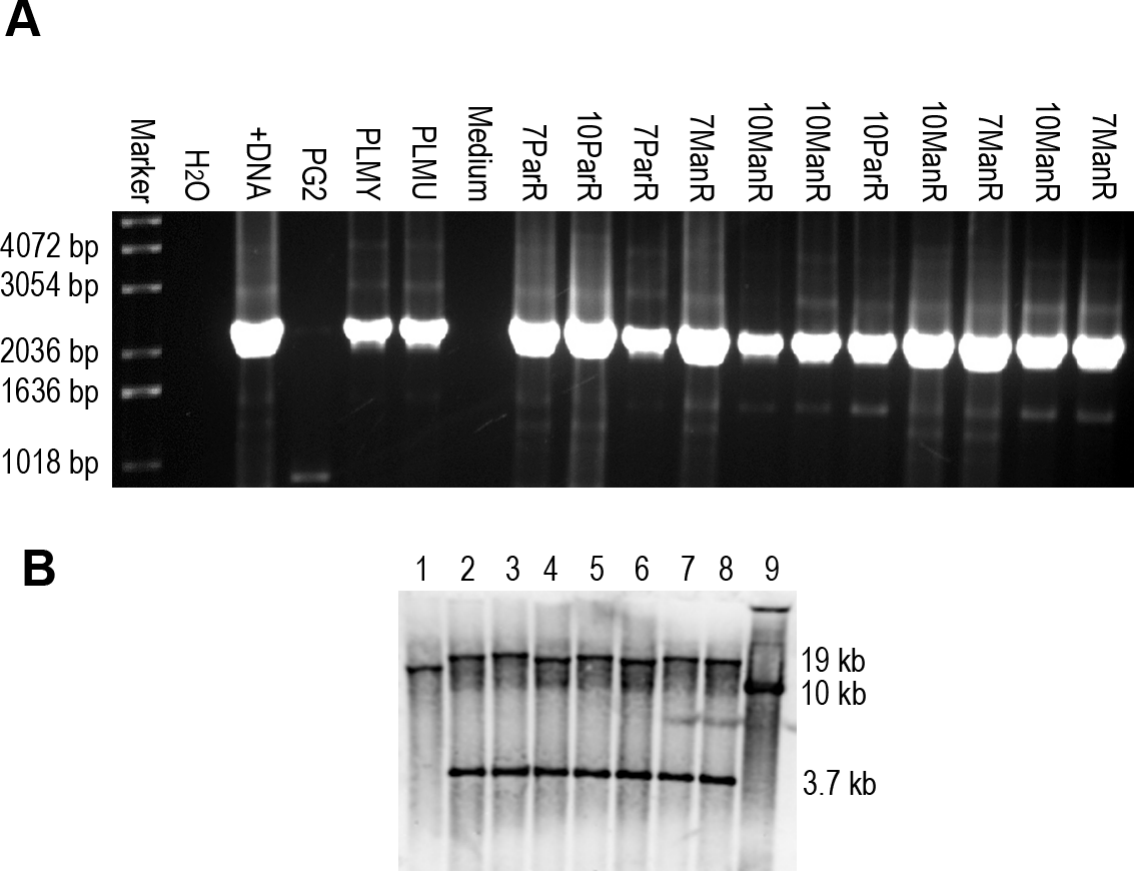
Confirmation of *xer1* disruption in selected *M*. *agalactiae* ‘switchover’ clones *via* PCR and Southern analysis. (A) Verification of *xer1* disruption in crude DNA extracts of selected ‘switchover’ clones by PCR using primers RecEndET28 and T3ISLrev specific to the chromosomal *xer1* region and the pR3 plasmid backbone, respectively. 2 kb PCR product confirms *xer1* disruption [13] in selected ‘switchover’ clones picked from the right (R)—parotideal (PAR) or—mandibular (MAN) lymph nodes of sheep MS 7 or MS 10. The observed bands were comparable to the positive controls corresponding to the crude DNA extracts (PLMY and PLMU) as well to pure DNA preparation of PLMY (+DNA); as expected this band was absent in the three negative controls corresponding to water (H_2_O), PG2 crude extract (PG2) and SP4 broth (Medium). (B) Southerns were performed as described earlier [13] whereby *Cla*I-digested genomic DNA was hybridized with *xer1*-specific probe. *xer1* disruption is confirmed in the ‘switchover’ clones (lanes 3–8) by the presence of two bands of 3.7 kb and ~18.9 kb as also seen for PLMU control (lane 2), whereas the wild type PG2 (lane 1) with intact *xer1* shows a 13 kb band. Disruption plasmid pR3 (lane 9) shows the expected 10 kb band. Switchover clones: lane 3, MS 10 right udder/VpmaV; lane 4, MS 6 mesenterial LN/VpmaW; lane 5, MS 6 right iliac LN/VpmaX; lane 6, MS 6 mesenterial LN/VpmaW **(PLM W);** lane 7, MS 9 right udder/VpmaX **(PLM X)**; lane 8, MS 6 right iliac LN/VpmaX.

Two mechanisms were considered to explain the unexpected altered Vpma phenotypes in many of the mycoplasma reisolates from the PLM-inoculated sheep: reversion of the *xer1* mutation or occurrence of *vpma* gene rearrangements in absence of Xer1. Disruption of the *xer1* gene was confirmed in the ‘switchover’ clones by PCR (Fig 5A) and Southern hybridization (Fig 5B) demonstrating the presence of the pR3/*tetM* disruption plasmid within *xer1* gene as described earlier for PLM construction [13]. Briefly, unlike the WT PG2 strain that displays a native 13 kb fragment in Southern analysis (Fig 5B, lane 1), pR3 integration at the chromosomal *xer1* locus results in duplication of the partial *xer1* sequence that segregates onto two *Cla*I fragments, one carrying the plasmid *oriC* region and part of the C-terminal coding region of *xer1*, and the other bearing the *bla* and *tetM* plasmid sequences together with the N-terminal region of *xer1*, followed by the *vpma* genes (Fig 5B, lanes 3–8).

### Complex *vpma* gene rearrangements led to gene duplications, deletions and chimeras causing altered Vpma phenotypes in ‘switchover’ clones

As VpmaW and VpmaX were the most predominant new alternative Vpma phenotypes selected *in vivo via* the generation of PLMW and PLMX clones, we decided to clone and sequence the whole *vpma* locus of two selected representative clones, namely PLM16 (PLMW) and PLM18 (PLMX) that constitutively express VpmaW and VpmaX, respectively (Fig 4). PLM16 was picked and filter cloned from the mesenterial LN of sheep MS 6 reisolates showing 100% VpmaW expression and the complete absence of the other five Vpma phenotypes (S1 Fig). PLM18, on the other hand, was picked from the predominant VpmaX expressors found in the right udder of MS 9.

**Fig 6:**
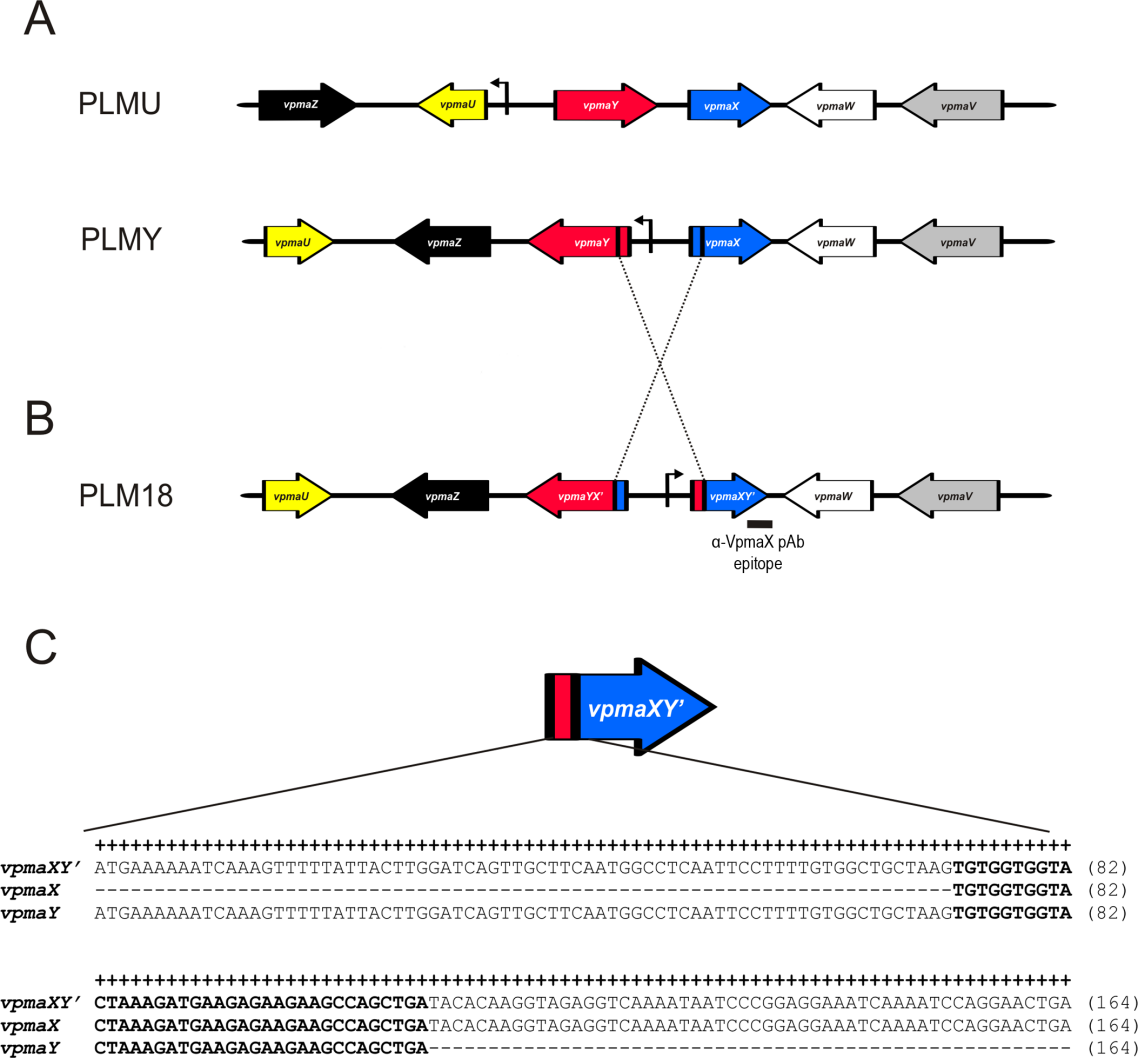
Schematic representation of the generation of hybrid *vpma* genes in PLM18 exhibiting VpmaX phenotype. (A) *vpma* configuration of PLMY and PLMU constituting the initial inoculum used for experimental infection of sheep: *vpma* genes are illustrated as large arrows in different colours, the unique promoter is shown as a bent arrow. (B) DNA sequence analysis of *vpma* configuration of PLM18 isolated from animals experimentally infected with an equal mixture of PLMY and PLMU: inversion of the promoter (crossed broken lines) and the generation of two hybrid *vpma* genes, *vpmaYX’* and *vpmaXY’* (indicated in blue/red colours), is illustrated. The *vpmaXY’* gene constitutes the expressed gene located downstream of the *vpma* promoter, whereas the *vpmaYX’* gene is transcriptionally silent. The VpmaX phenotype of PLM18 can be explained by the fact that the coding sequence corresponding to the epitope recognized by α-X pAb is present at the 3’ end of the expressed *vpmaXY’* gene (black bar). (C) Sequence alignment of the hybrid *vpmaXY’* gene of PLM18 with the sequence of the original *vpmaX* and *vpmaY* genes: a 38-bp region common to both *vpma* genes (bold letters) is identified in the hybrid *vpmaXY’* gene. The upstream region of the common 38-bp region in *vpmaXY’* shows complete sequence identity with the *vpmaY* gene, whereas the downstream region is identical with the *vpmaX* gene, clearly indicating that the homologous 38-bp region was involved in an intergenic recombination event between *vpmaX* and *vpmaY*, which led to promoter inversion and generation of hybrid *vpmaXY’* gene.

The *vpma* configuration of PLM18 was found to be similar to PLMY (Fig 6A) except that two hybrid genes, *vpmaYX’* and *vpmaXY’* are present instead of *vpmaX* and *vpmaY* genes of the type strain PG2. Located downstream to the single identified promoter, *vpmaXY’* constitutes the expressed gene in PLM18 (Fig 6B). Comparison of the coding sequence of the hybrid *vpmaXY’* gene with the original *vpma* sequences of the PG2 clonal variant 55–5 [24] and PLMY [13] (Fig 6A) indicates that an intergenic recombination event has occurred at a 38-bp sequence (Fig 6C, bold letters) that is common to both *vpmaX* and *vpmaY* gene sequences. In the chimeric gene *vpmaXY’*, the sequence upstream of this 38-bp homologous region was found to be 100% identical with the *vpmaY* gene, whereas the downstream region showed complete identity with the sequence *vpmaX* (Fig 6C). Recombination at this 38-bp homologous sequence not only resulted in the generation of two chimeric genes, but also led to the inversion of the single promoter resulting in the alteration of the observed Vpma phenotype depending on the specific Vpma antibody epitopes present in the downstream region (Fig 6B).

**Fig 7:**
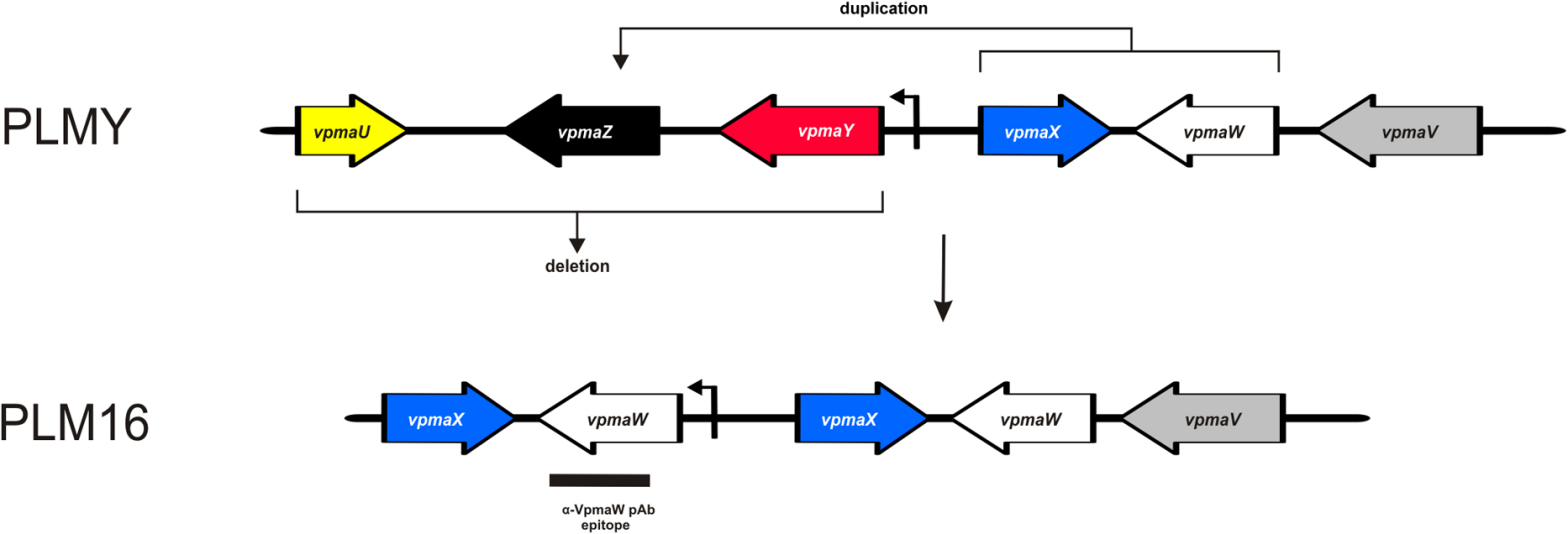
Schematic depiction of gene duplications and deletions in the *vpma* locus of PLM16 expressing VpmaW. DNA sequence of the *vpma* locus revealed the presence of the *vpmaW* gene downstream of the promoter (bent arrow), which correlates with the VpmaW phenotype of PLM16. DNA-sequence analysis revealed the duplication of *vpmaX* and *vpmaW*, whereas *vpmaY*, *vpmaU* and *vpmaZ* are deleted. The coding sequence corresponding to the epitope recognized by α-W pAb is shown as a black bar below the *vpmaW* gene. Potential gene duplication and deletion events, which likely occurred in PLMY and led to the *vpma* configuration of PLM16 are illustrated.

**Fig 8:**
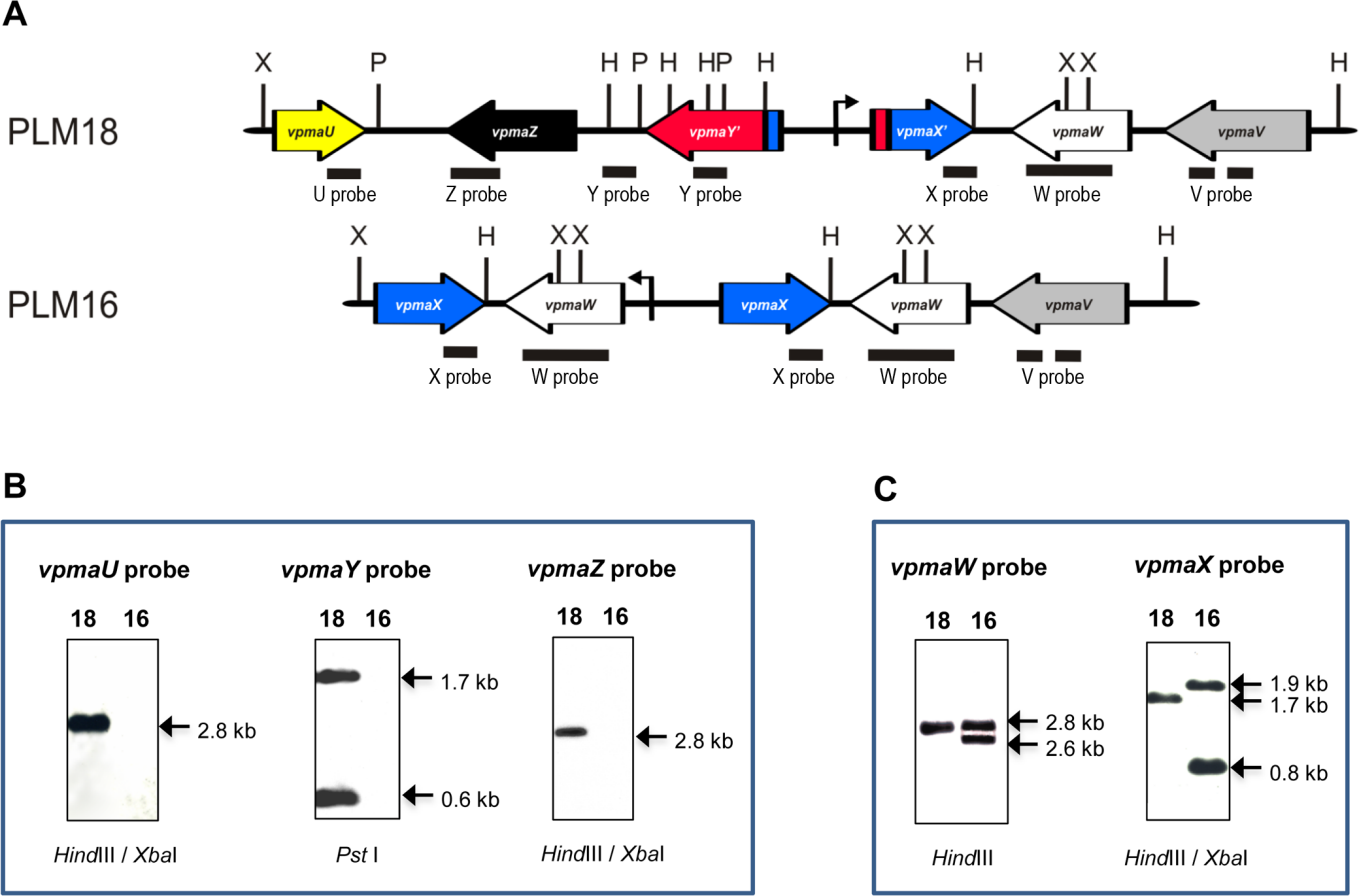
Confirmation of PLM16 (PLMW) and PLM18 (PLMX) *vpma* gene repertoire by Southern analysis. (A) Restriction maps depicting the binding sites (black boxes) of *vpma*-specific gene probes within the *vpma* gene loci of PLM18 and PLM16: H, *Hind*III, P, *Pst*I; X, *Xba*I. (B) Southern blots demonstrating the absence of *vpmaU*, *vpmaY* and *vpmaZ* in PLM16 compared to PLM18, which shows the expected bands of 2.8 kb (with *vpmaU*- and vpmaZ-specific probes) and, 1.7 and 0.6 kb (with *vpmaY*-specific probe) (C) Southern blots confirming the duplication of *vpmaW* and *vpmaX* genes in PLM16 compared to single copies of these in PLM18, visible as 2.8 kb and 1.7 kb bands, respectively. PLM16 shows a doublet of 2.8 and 2.6 kb with *vpmaW* probe, whereas *vpmaX* probe identifies the expected 1.9 kb and 0.8 kb *Hind*III/*Xba*I fragments. Restriction enzymes used for genomic DNA digestions of PLM16 (16) and PLM18 (18) for each Southern are indicated below the respective blots.

In contrast to PLM18, examination of the PLM16 *vpma* locus revealed a much more complex recombination scenario. Phenotypic expression of VpmaW in this clone correlates well with the sequence analysis that revealed the presence of *vpmaW* gene located downstream of the promoter (Fig 7). Sequencing data also revealed the duplication of two *vpma* genes, namely *vpmaX* and *vpmaW*, as also supported by Southern blot analysis whereby two bands were observed with both *vpmaW* and *vpmaX* probes when the genomic DNA was digested with restriction enzymes that cut outside these genes, respectively (Fig 8A and 8C). Furthermore, the sequence of PLM16 *vpma* locus also showed the complete absence of *vpmaU*, *vpmaY* and *vpmaZ* genes (Fig 7). In accordance, no hybridization signal was observed during Southern blot analysis with probes specific for *vpmaU*, *vpmaY* and *vpmaZ* genes (Fig 8B) clearly verifying the absence of these three genes. In comparison, PLM18 demonstrated the expected bands with the specific *vpma* gene probes, for instance, 2.8 kb *Hind*III or *Hind*III/*Xba*I fragments corresponding to *vpmaU*, *vpmaW* and *vpmaZ* genes, respectively (Fig 8B and 8C). A 1.7 kb *Hind*III fragment corresponds to the *vpmaX*-derived sequence of the hybrid *vpmaYX’* gene in PLM18, whereas the presence of two *Hind*III/*Xba*I fragments (0.8 kb and 1.9 kb) indicates duplication of *vpmaX* in PLM16 (Fig 8C). Similarly, *Pst*I-digested genomic DNA probed with a *vpmaY*-specific probe detects 0.6 kb and a 1.7 kb fragments corresponding to the *vpmaY*-derived sequence of the hybrid *vpmaYX’* gene in PLM18 and both these fragments are absent in PLM16 (Fig 8B).

Although, it is difficult to precisely reconstruct the hierarchy of genomic rearrangements that occurred in PLM16, the final *vpma* configuration of this clone (Fig 7) is likely a result of recombination events that occurred in PLMY. Potential duplication of *vpmaX* and *vpmaW* placed the *vpmaW* gene downstream of the promoter, whose position is unaltered compared to PLMY, and simultaneously deleted the genomic fragment carrying *vpmaU*, *vpmaY* and *vpmaZ* (Figs 7 and 8).

## Discussion

Pathogenicity is a complex summation of many biological processes that allow the pathogen to survive, multiply and persist in an immunocompetent host. This is especially true for mycoplasmas that usually cause highly persistent infections without being recognized by host immune factors and are thus considered useful models to investigate transitions from a parasitic to endosymbiotic life style [25]. While their pathogenicity mechanisms are largely unknown, persistence is often attributed to the presence of large multigene families causing high-frequency phase variation of surface lipoproteins [1, 4, 5]. This study provides the first experimental in-host demonstration of their significance and uncovers new facets of mycoplasma antigenic variation systems.

The biological processes relevant to pathogenicity are often regulated by a complex spectrum of different host and microbial molecules, stressing the need for more relevant *in vivo* studies, without which, the *in vitro* models would be largely incomplete, and sometimes even misleading [26]. This further highlights the relevance of our study as it was carried out in the natural host using two different natural routes of infection.

### Vpma phase variation is important for in-host survival of the pathogen

*M*. *agalactiae* PLMs constitutively and stably express the same defined Vpma protein *in vitro* and are unable to undergo further switching as their *xer1* gene responsible for Vpma phase variation is disrupted [13]. Yet, when tested in intramammary and conjunctival sheep infection models, PLMY and PLMU, initially expressing just VpmaY and VpmaU, respectively, were able to express new Vpmas after in-host passage. (Tables 1 and 2; Fig 2; S1 and S3 Figs). The in-host selection of these ‘switchovers’, previously undetected *in vitro*, indicate that these display a phenotypic advantage. Furthermore, these ‘switchovers’ behaved like new PLMs and showed no further phase variations for the rest of the experiment *in vitro* (Fig 4). This finding demonstrated the occurrence of an alternative Xer1-independent mechanism of Vpma switching and strongly emphasizes the significance of Vpma antigenic variation for *M*. *agalactiae*. Similar alternative *in vivo* switching of surface layer proteins (SLP) has also been reported for a *recA-*disrupted PLM of *Campylobacter fetus* during experimental sheep infections [27]. However, unlike our study, in this case the two PLMs used for infection predominantly did not switch and just one of the ovine–passaged isolates switched to the other mutant SLP phenotype without creating new SLP variants [27, 28].

### Complex rearrangements in the *vpma* gene repertoire of *M*. *agalactiae in vivo*

In-host change of Vpma expression by PLMs was a result of complex *vpma* gene rearrangements that differed from conventional Xer1-mediated site-specific *vpma* recombinations. In one of these switchover clones, three of the six *vpma* genes were completely missing and two others were duplicated (Figs 7 and 8). In another clone (Fig 6), intergenic *vpma* recombination at a 38-bp homologous region generated new chimeric *vpma* genes that resulted in changed Vpma phenotype of the PLM. This is remarkable in many aspects. Firstly, the likelihood of a recombination occurring at this short homologous sequence is rare and emphasizes the strong *in vivo* selection pressure faced by the PLMs to change their Vpma phenotype. This contrasts the *in vitro* scenario where PLMs never changed Vpma expression [13], and generally speaking, targeted gene manipulations *via* homologous recombination are still a challenge for mycoplasmologists [12]. The presence of several intergenic and intragenic repeat sequences in the *vpma* locus offers an enormous potential for generating many new hybrid Vpma antigens *via* recombination *in vivo*. Repeated regions are also found in the phase variable loci of other mycoplasmas and the generation of hybrid genes by homologous recombination likely represents a common mechanism for additional antigenic diversification for immune evasion in these species. For instance, similar intrachromosomal recombination events in the *vsp* gene locus of *M*. *bovis* led to the generation of a new chimeric lipoprotein [29]. Vpma hybrids have been reported in strain 5632 of *M*. *agalactiae*, which incidentally contains an extended repertoire of 23 *vpma* genes distributed on two separate *vpma* loci. However, the occurrence of a second *vpma* cluster at a separate locus is a rare event, so far observed only in strain 5632 and found absent in all the 92 different *M*. *agalactiae* strains tested [30]. Yet our study provides the first demonstration that such variations are a result of in-host selective pressure, in the absence of which the pathogen maintains its basic *vpma* genotypic switching *via* site specific recombination [13, 14, 20]. A possible explanation for this could be the better fitness of these classical “simple variants” in the absence of immune responses as demonstrated for Msp2 antigenic variants of *Anaplasma marginale* [8].

### Vpma phase variation plays a role in host immune evasion

Among various strategies used by pathogens to persist in the immunocompetent hosts, evasion of host defence reactions by antigenic variation is quite significant [8, 31, 32]. However, in mycoplasmas, which are known for their sophisticated phase-variable surface protein families, the function of these proteins in immune evasion in the host has not been unequivocally demonstrated [5, 6].

For *M*. *agalactiae*, this issue was unexplored until now although Vpma immunogenicity and *in vivo* phase variations have been demonstrated [23]. Since the Vpma switching rates are very high [13] it was not possible to reliably assess the role of Vpma phase variation in host colonization and immune evasion during *in vivo* studies with the wild type PG2 strain. The PLMs offered this opportunity, and when used in the sheep infection model they exhibited Xer1-independent complex ‘switches’ that were observed only in the immunocompetent host and never during several *in vitro* passages. From this we envisaged that these Vpma switches are likely involved in host immune evasion. In *Anaplasma marginale*, “complex variants” of Msp2 are favoured only under selective pressure of adaptive immune response as they generally have a reduced fitness compared to the “simple variants” [8]. Similar competing selection pressures for immune evasion and variant fitness might also apply to the Vpma variants obtained by conventional Xer1-mediated and complex Xer1-independent recombination. Furthermore, it has been shown in *Campylobacter fetus* that host immune responses against the antigenic SLPs are delayed in sheep infected with strains capable of varying their SLPs, as compared to those infected with SLP phase-invariable mutants [22]. Although mycoplasma lipoproteins are well-known preferential targets of the humoral immune response, yet the influence of host antibodies on mycoplasma phase-variable lipoproteins, including Vpmas, has never been directly investigated *in vivo* [5].

In the current study, VpmaU- and VpmaY-specific antibody responses in milk and sera of the PLM-infected sheep (Fig 3) correlate strongly with the presence of specific Vpma expressors at different phases of infection, and with the appearance of ‘switchover’ clones in PLMs (Tables 1 and 2). This suggests that Vpma switching may be a key factor of *M*. *agalactiae* persistence.

### Differential infection potential of Vpma phase variants

VpmaY expressors (PLMY) clearly exhibited a better fitness *in vivo* compared to VpmaU expressors (PLMU) (Tables 1, 2, 3 and 4; Fig 3). This is a very interesting result which could have a great impact on our understanding of Vpma-host interactions. Growth *in vitro* is very different than growth *in vivo*, and the latter is a primary requisite for pathogenesis [33]. As PLMU did not show any *in vitro* growth retardation, this is an exclusive *in vivo* characteristic likely dependent on one or more host factor interactions. Lipoproteins of bacterial pathogens are known to be involved in host adaptation and immune evasion, and can act as cytadhesins, invasins, potent immunogens and immune modulators by interacting with the innate and adaptive host immune responses [6, 34]. Although unknown at this point, VpmaY and VpmaU lipoproteins could interact and likely differ with each other at any of these levels. It is possible that VpmaY being longer in size has a better ‘shielding’ capacity against phagocytic cells as observed for Vsa lipoproteins of *M*. *pulmonis* [11], or perhaps it enhances adherence of the organism or even the retrieval of nutrients for growth and multiplication resulting in a better survival or *in vivo* growth rate.

*M*. *agalactiae* infections initiate a dynamic innate cellular response, followed by a chronic adaptive response [35, 36]. Mycoplasma lipoproteins being potent macrophage stimulants are often implicated in the initiation of a characteristic immunopathology [6]. Hence, considering the clearance of PLMU right at Day 1 p.i., it is likely that the VpmaU lipoprotein either hindered initial colonization, perhaps due to lower cytadhesion ability compared to VpmaY, or it made the mycoplasma cells more vulnerable to early immune responses. However, all this needs further investigations, which are currently underway.

Furthermore, the *in vivo* selection and enrichment of VpmaW and VpmaX expressors is also noteworthy as these were the only Vpma phenotypes detected exclusively in some tissues (Fig 2, Table 2, S1 Fig), which might be indicative of some preference or tendency for tissue tropism though not detected in all sheep and would require further evaluations. A similar *in vivo* enrichment of specific VlhA lipoprotein isotypes of *M*. *gallisepticum* was observed in multiple independent hosts during experimental chicken infections [10]. This in-host expression and enrichment of specific Vpma phenotypes is indicative of their importance in host-pathogen interactions. Interestingly, VpmaX and VpmaW were also observed to be most immunogenic amongst the Vpma proteins when we tested the serum from a convalescent naturally infected ewe (PAL 97) [37] against the corresponding six MBP-Vpma fusion proteins [13] (S4 Fig).

### Vpma expression at different stages of infection

Requirements for virulence factors are known to change with different infection models and also as the infection proceeds. It has been reported that in some pathogens a particular phase variant, or even phase variation *per se*, is required only at a particular infection stage and not throughout the entire infection process [38, 39]. This may as well apply to Vpmas and their phase variation during sheep infections. Although PLMU was cleared in the beginning, PLMY could successfully establish local infection even in the absence of Vpma phase variation. But, at later stages of infection, likely to cope with host defence and to persist, or to become systemic, it was necessary for the organism to vary the Vpma phase, and hence the selection pressure that induced Xer1-independent alternate ‘switches’ in PLMs. This is supported by an earlier study where PG2 type strain induced better systemic responses and also disseminated better in infected sheep compared to PLMs [21], likely by delaying the host anti-Vpma antibody response by varying the Vpma antigens as observed in case of SLP antigenic variation of *Campylobacter fetus* [22]. Also, considering that Vpmas have adherence epitopes [20], their differential adherence rates might also govern the selection of different Vpma variants at specific stages of local or systemic infections as observed in other pathogens [40].

### Conclusion

Our results strongly emphasize the in-host significance of Vpma rearrangements for *M*. *agalactiae*. Although Xer1 recombinase is the sole factor responsible for Vpma switching *in vitro* in the absence of any discriminatory constraints, alternative molecular switches become prominent in its absence under selective pressure as observed inside the immunocompetent sheep host mounting Vpma-specific humoral responses. Furthermore, these host responses are instrumental in selecting complex novel antigenic variants, a causal relationship though known in other pathogens [8] was so far not shown for *M*. *agalactiae*. This is the first report where mycoplasma PLMs have been used for infection studies in the natural host pointing towards the differential pathogenicity of such phase variable lipoproteins and providing new insights into the mechanisms of their antigenic variation inside an immunocompetent host during disease progression. This will likely impact the understanding of the *in vivo* role of surface antigenic variation systems and their regulation in other pathogenic mycoplasmas. Enhancing our understanding of the fitness costs of specific expression variants might also make it feasible to develop new vaccines that prevent the period of high infectivity and disease by targeting highly fit clonal variants [32].

## Materials and methods

### Infection inoculum

Amongst the six Vpma lipoproteins expressed by the *M*. *agalactiae* type strain PG2, VpmaY and VpmaU are not only the most abundant variants observed *in vitro*, but also, based on N-terminal and other repeat sequences, belong to the two separate homology groups identified by Glew *et al* [20]. Hence, PLMY and PLMU, constitutively expressing VpmaY and VpmaU respectively [13] were selected as representatives for sheep infection trials to check their comparative *in vivo* behavior. *M*. *agalactiae* pathogenic type strain PG2 [41] was used for inoculating the positive control group. The type strain and PLMs were grown in Aluotto broth [42] and processed for inoculum preparation as described earlier in detail [21]. Unlike the phase-invariable PLMU and PLMY, the PG2 population expressed all six Vpmas with high-frequency phase variations [13]. The Vpma phenotypes were reconfirmed by plating and re-analysing the residual pooled inocula using Vpma-specific antibodies, whereby the PLM inoculum was confirmed to contain only VpmaY and VpmaU expressors and the other four Vpma phenotypes were not detected.

### Animals and experimental infections

Details of the experimental set-up of the sheep infection trials have been reported earlier [21] and the main points enlisted under Table 5. Briefly, for both the intramammary and the conjunctival infection routes, 5 sheep were used for each of the three infection groups (lambs, denoted by the initial ‘S’, for conjunctival infections, and lactating ewes, denoted by ‘MS’, for intramammray infections): (i) the experimental PLM group infected with an equal concentration mixture of PLMU and PLMY (5 x 10^8^ cfu, each), (ii) a positive control group infected with 10^9^ cfu of the PG2 strain, and (iii) a negative control group inoculated with pyrogen-free saline (see Table 5). Animal samples, such as blood, milk and ocular and nasal swabs were collected regularly as described earlier [21], always proceeding from the negative control group to the PLM-infected sheep, followed by the positive control group at the end. The samples were processed further for storage at -80°C or for immediate analysis. Sheep were euthanized and necropsied at Day 20 p.i. (conjunctival infection) or Day 28 p.i. (intramammary infection). Samples from organs (spleen, lungs, kidneys and udders) and LNs (such as mandibular, mediastinal, mesenterial, medial and lateral retropharyngeal, parotideal, iliac, supramammary, etc) were cut-up into small pieces and flash-frozen in liquid nitrogen before storing at -80°C.

**Table 5:** Basic configuration of the experimental *M*. *agalactiae* infection trials.

Groups	Negative control	Phase-locked mutants	Positive control
**Infection material**	PBS	*M*. *agalactiae* Vpma-phase locked mutants: PLMU & PLMY	*M*. *agalactiae* PG2
**Inoculum**	-	5 x 10^8^ each mutant (total 10^9^)/sheep	10^9^/sheep
**Sheep Numbers**	Conjunctival infection: S1—S5 or Intramammary infection:MS1—MS5	Conjunctival infection: S6 –S10 or Intramammary infection:MS6 –MS10	Conjunctival infection:S11 –S15 or Intramammary infection:MS11- MS15

### Ethics statement

The sheep conjunctival and intramammary route infections were performed with the approval of the Austrian Federal Ministry for Education, Science and Culture (BMBWK-68.205/0145-BrGT/2006) and the Austrian Federal Ministry for Science and Research (GZ/BMWF-68.205/0092-C/GT/2007), respectively. All procedures related to the sheep experiment were carried out according to the Animal Experiments Act (TVG, BGBI.Nr. 501/1989, last modified by BGBI. I Nr. 162/2005). The animals were housed in the stables at the University of Veterinary Medicine Vienna and experiments executed after approval by the Ethics and Animal Welfare Commission of the University of Veterinary Medicine Vienna. The sheep were anesthetized by Thiopental before euthanizing them *via* intravenous injection of T61 as recommended and approved for sheep according to the drug directory of the Austria Codex.

### Vpma phenotyping of mycoplasma reisolates from sheep tissues, milk and swab samples

Original or diluted frozen stocks corresponding to the samples found positive for *M*. *agalactiae* in a previous report [21] were subjected to analysis for Vpma phenotyping. Undiluted or appropriate serial dilutions of the stocks were directly plated on Aluotto or SP4 agar plates and incubated at 37°C for a minimum of 7 days. The frozen tissues were also re-analyzed by cutting-off small pieces, finely chopping and resuspending in 3–5 ml Aluotto broth, which was incubated at 37°C for 3 h before plating. Agar plates with an appropriate number of well-isolated colonies were selected for Vpma phenotyping *via* colony immunoblot analysis.

Colony immunoblotting was performed essentially the same way as described earlier [13]. Briefly, the colonies were lifted on Protran BA 83 nitrocellulose membranes (Schleicher & Schuell), allowed to dry at room temperature (RT) and rinsed 3 times in TBS buffer (10 mM Tris, 154 mM NaCl, pH 7.4) before incubating overnight at 4°C in 1: 2000 dilution of VpmaY-specific α-Y or 1: 600 dilution of VpmaU-specific α-U rabbit polyclonal antisera [13]. Membranes were washed three times with TBS buffer containing 0.05% Tween 20 (Roth) for 10 minutes each with shaking. Subsequent incubation in swine anti-rabbit IgG conjugated to horseradish peroxidase (Dako) was carried out at least for 1 h at RT at 1: 2000 dilution. The membranes were then washed three times for 10 min each in TBS buffer before developing them in 4-chloro-1-naphthol (Bio-Rad) and hydrogen peroxide for 15–30 min. Finally, the blots were rinsed in water, dried at RT and viewed under a Nikon SMZ-U stereomicroscope to count the number of positive colonies among the total colonies present on the blot. If needed, the negative colonies were counterstained in pink color using reversible Ponceau S (Roth) staining to calculate the percentage of PLMU or PLMY clones in the total mycoplasma load of the specific sample.

After the initial analysis with α-Y or α-U Abs, the expression of the other four Vpmas, namely VpmaW, VpmaX, VpmaZ and VpmaV was also checked in the samples *via* similar colony immunoblot analysis using α-W, α-X, α-Z and α-V rabbit polyclonal Abs [13], respectively.

### PLMY and PLMU mixed culture growth assay

Two different -80°C stocks containing the PLMU and PLMY mixture for sheep inoculations were thawed. The average mycoplasma count had been earlier calculated (by thawing and plating of two other independent aliquots) to be 3.4 x 10^10^ cfu/ml. The thawed stocks were gently vortexed before removing 70 μl aliquots to inoculate 25 ml Aluotto broth containing penicillin and phenol red as described earlier [43]. Dilutions (10^−4^ to 10^−6^) of the duplicate cultures were plated on SP4 and Aluotto agar at time T0 of the growth assay before incubation at 37°C. Further 100 μl samples were removed after 3, 5, 23, 31, 48 and 70 h of incubation to prepare duplicate serial dilutions (10^−4^ to 10^−7^) for plating 100–200 μl on Aluotto and SP4 agar plates in duplicate. The plates were incubated at 37°C for 5–7 days. Colonies were counted under the Nikon SMZ-U stereomicroscope to calculate the final cfu/ml, and for each time point, plates with an appropriate number of well-isolated colonies were used for colony immunoblot analysis. Each of the duplicate colony blots was cut into two equal halves, one half was immunoblotted with an α-U Ab specific for VpmaU and the other half with α-Y antiserum specific for VpmaY. In each case, positive colonies were counted under the stereomicroscope before counterstaining the negative colonies with Ponceau S to calculate the proportion of PLMU and PLMY in the total population. Absence of the other four Vpmas in this PLMU and PLMY mixed culture was confirmed by colony immunoblot analyses with α-W, α-Z, α-V and α-X antisera, all of which gave negative results.

### Western blot analyses of sheep milk and sera

A Mini-PROTEAN II multiscreen apparatus (Bio-Rad) was used to screen the individual sheep sera and milk samples on Western blots. For antigen preparation, strain PG2, PLMY and PLMU were grown in 50 ml Aluotto broth for 3–4 days at 37°C before centrifugation at 10,000 g for 15 minutes. The cell pellets were resuspended in 1–2 ml PBS, and the protein concentrations were determined using the Pierce BCA Protein assay kit (Thermo Fisher Scientific). About 300–400 μl of each of the cell suspensions was separately loaded onto 12% polyacrylamide gels containing 3% (w/v) urea for running standard SDS-PAGE mini gels which were blotted onto Protran nitrocellulose membranes as described earlier [13]. The blots were blocked with 3% (w/v) skimmed milk and rinsed in TBS before applying to the multiscreen apparatus. Milk and sera samples were diluted 1:100 and 600 μl of each was used per channel of the multiscreen apparatus. After overnight incubation at 4°C, the blots were processed using the same protocol as described for colony immunoblotting except that polyclonal rabbit anti-sheep immunoglobulins (Dako) were used as secondary antibodies at a dilution of 1: 2000.

### Selection, filter cloning and characterization of new PLMs

Many of the biological samples obtained from PLM (PLMU and PLMY)-infected sheep revealed colonies expressing Vpma phenotypes other than VpmaU and VpmaY. Colony immunoblots with α-V, α-X, α-Z and α-W antibodies revealing predominantly positive colonies were used to pick the corresponding clones (expressing VpmaV, VpmaX, VpmaZ and VpmaW, respectively) from the agar plates under the microscope, transferred into 1 ml SP4 broth containing tetracycline (2 μg/ml) and grown for 5–7 days at 37°C. A small aliquot was then used for making serial dilutions to be plated for colony immunoblot analysis with Vpma-specific antibodies, and the rest was used to extract crude DNA,which was tested for *xer1* disruption by PCR using primer RecEndET28, corresponding to the chromosomal *xer1* gene, and primer T3ISLrev, corresponding to the plasmid backbone of the disruptant plasmid as described earlier [13]. Once the *xer1* disruption was confirmed with the presence of a 2 kb band, additional colony immunoblots were analysed with the six Vpma-specific antibodies. As the picked colonies were positive only with a single Vpma-specific antibody (and negative for all the other five Vpmas) without any sectoring phenotype, we concluded that these ‘switchover’ Vpma expressors were new PLMs, now expressing Vpma phenotypes other than VpmaU and VpmaY. For each of these new PLMs, namely PLMV, PLMX, PLMZ and PLMW, filter cloning was performed using three rounds of plating, colony immunoblotting and isolating single colonies. Briefly, cells from a colony (corresponding to a positive colony immunoblot phenotype as visualized by a specific Vpma Ab) were picked using a sterile micropipette, transferred into 1 ml SP4 medium and vortexed. The cell suspension was then sucked into a syringe with a 0.9 mm needle and filtered successively through 0.45 μm and 0.2 μm disposable membrane filters. Dilutions (10^−1^ to 10^−3^) were made from both filtrates, and 200 μl of each dilution was plated on SP4 agar plates containing tetracycline (2 μg/ml). The plated mycoplasma cells were incubated at 37°C until colonies were visible, and another round of colony immunoblotting was made to again pick single well-isolated positive colonies from corresponding agar plates. This procedure was repeated once again, and at the end, the new PLMs were thoroughly characterized with all six Vpma-specific Abs and also checked for the *xer1* gene disruption as described above. Southern blot analyses for verification of *xer1* disruption and *vpma* gene configuration are described ahead under separate section headings.

### Confirming *xer1* disruption *via* Southern blot analyses

Mycoplasma genomic DNA was isolated by QIAamp DNA Mini Kit (Qiagen) and digested with *Cla*I (New England Biolabs) at 37°C for at least 6–7 h before subjecting to electrophoresis on a 1% agarose gel. This was followed by standard Southern blotting procedures and hybridization using Digoxigenin (DIG)-labelling system (Roche) as described previously [43]. A DIG-labelled probe corresponding to a 513 bp partial *xer1* fragment was prepared by PCR amplification of genomic DNA of type strain PG2 using primers XerR and XerS in presence of 2.5 mM MgCl_2_ using 30 cycles of 95°C for 43 s, 56°C for 43 s and 72°C for 43 s. After purification with the QIAquick PCR Purification Kit (Qiagen), hybridization and subsequent chemiluminescent detection of the DIG-labelled nucleic acids using Anti-DIG -AP was carried out according to the manufacturer’s instructions (Roche). As described earlier [13], *xer1* disruption corresponded with the presence of two disruption bands of about 3.4 kb and 18.9 kb, whereas the wild type PG2 strain yielded a 13 kb band under these conditions.

### Cloning and sequencing of *vpma* loci

In order to define the *vpma* configuration of selected ‘switchover’ clones, such as PLM16 and PLM18, their *Cla*I-digested genomic DNA was self-ligated and transformed into *E*. *coli* DH10B. Tetracycline- and ampicillin-resistant transformants were selected and recombinant plasmids, namely pPLM16 and pPLM18, were isolated from them using the EZNA Plasmid Miniprep Kit DNA (Peqlab). The obtained plasmid DNA was used for sequencing the *vpma* gene loci present in these clones using the primer walking approach. Sequencing and synthesis of all oligonucleotides used in this study (see S1 Table) was carried out at LGC Genomics GmbH, Berlin, Germany. Sequences were analyzed by advanced BLASTX searches made at the website for the National Centre for Biotechnology Information: https://blast.ncbi.nlm.nih.gov/Blast.cgi.

### Attestation of *vpma* gene configuration *via* Southern analyses

DIG-labelling (Roche) of *vpma*-specific gene probes by PCR was carried out according to manufacturer’s instructions using PG2 genomic DNA as template and a set of following primer pairs (oligonucleotide sequences are enlisted in S1 Table) for individual *vpma*-specific gene probes: U2F and Urev1 (*vpmaU*), WDIGfw and WDIGrv (*vpmaW*), X1F and X1R (*vpmaX*), Y3F and Y3R (*vpmaY*), Z1F and Z2R (*vpmaZ*). PCR cycling conditions were as follows: 1 cycle of initial denaturation for 3 min at 94°C, 30 cycles of 95°C for 1 min, 57°C (*vpmaU*, *vpmaX*, *vpmaY*, *vpmaZ*) or 65°C (*vpmaW*) for 1 min, followed by 30s (*vpmaU*, *vpmaX*, *vpmaY*, *vpmaZ*) or 1 min (*vpmaW*) at 72°C and a final extension step for 5 min at 72°C.

Mycoplasma genomic DNA was isolated as described previously [43]. For *vpma*-specific Southern blots, genomic DNA was digested with appropriate restriction endonucleases: *Hind*III for hybridization with *vpmaW*-specific probe, *Hind*III and *Xba*I for *vpmaU*-, *vpmaX*- and *vpmaZ*-specific probes and *Pst*I for *vpmaY-*specific probe. Digested DNA was subjected to agarose gel electrophoresis and DNA fragments were transferred to nylon membranes (Roth) using standard procedures described earlier [13]. Hybridization with DIG-labelled probes and washing under stringent conditions followed by non-radioactive detection was carried out according to the manufacturer’s recommendations (Roche).

## Supporting information

S1 Fig Representative colony immunoblots of *M*. *agalactiae* reisolates obtained from the mesenterial lymph node of sheep MS 6. Colonies were positive only when immunostained with VpmaW-specific antisera (α-W) and were negative with all the other five Vpma specific pAbs (α-U, α-Y, α-Z, α-X and α-V) and were counterstained in pink using the non-specific protein dye (Ponceau S).
(PDF)

S2 Fig Representative colony immunoblots of *M*. *agalactiae* reisolates obtained from right udder halves of sheep MS 9. All colonies were negative for immunostaining using VpmaY- and VpmaU-specific α-Y and α-U antisera, respectively, and appeared pink with the non-specific Ponceau S counterstaining.
(PDF)

S3 Fig Representative colony immunoblots of *M*. *agalactiae* reisolates obtained from right udder halves of sheep MS 8. Many colonies were positive when immunostained with α-U pAb and were completely negative with α-Y and α-Z, whereas singular positive colonies were observed with α-W, α-X and α-V antisera. Negative colonies were counterstained in pink using the non-specific protein dye (Ponceau S).
(PDF)

S4 Fig VpmaW and VpmaX are highly immunogenic and evoke a strong antibody reaction during natural sheep infections. (A) Expression of MBP (Maltose Binding Protein)-Vpma fusion proteins [13] as observed on Coomassie blue-stained reducing SDS-polyacrylamide gel. Z1 and Z2 correspond to two different (regions) VpmaZ-MBP fusion proteins. (B) Immunostaining with polyclonal anti-*M*. *agalactiae* serum PAL-97 obtained from a naturally infected sheep [37].
(PDF)

S1 Table Oligonucleotide sequences used in this study. (DOCX)
