# Supplementary figures and images for: Vpma phase variation is important for survival and persistence of Mycoplasma agalactiae in the immunocompetent host
Source: PLoS Pathog. 2017 Sep 28;13(9):e1006656. doi: 10.1371/journal.ppat.1006656 (PMC5634654; doi:10.1371/journal.ppat.1006656)

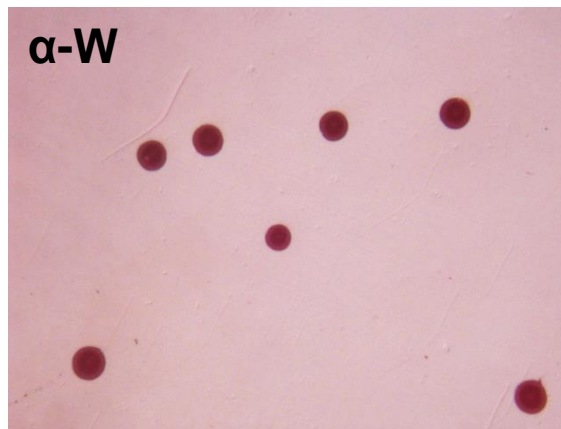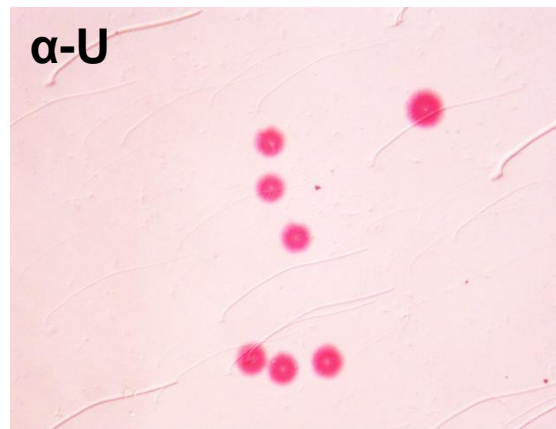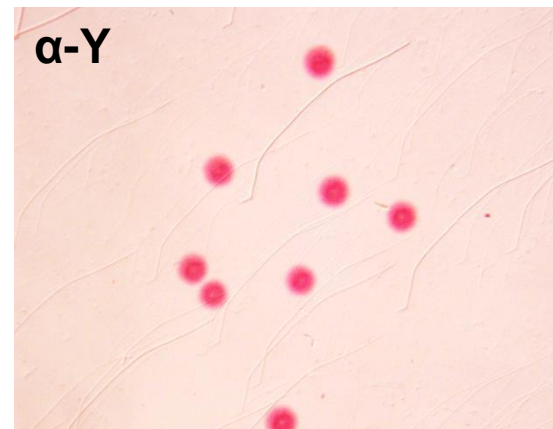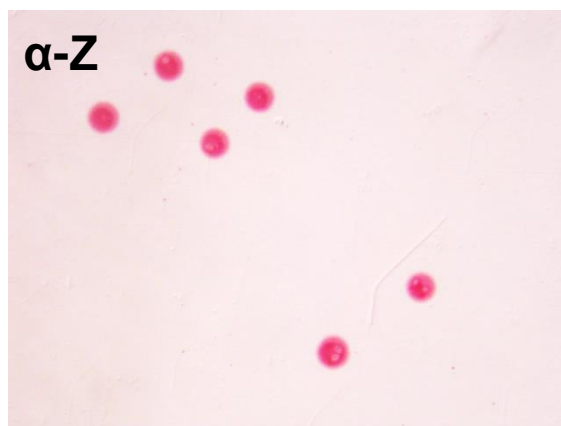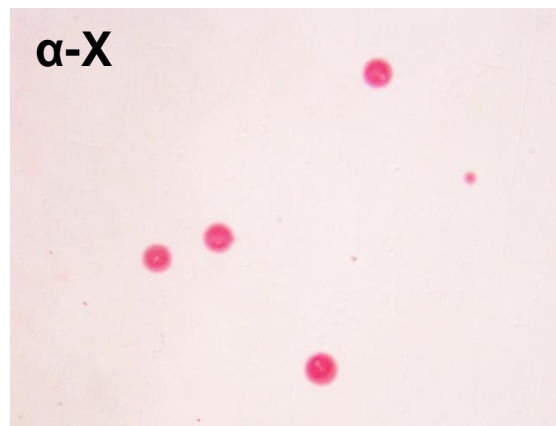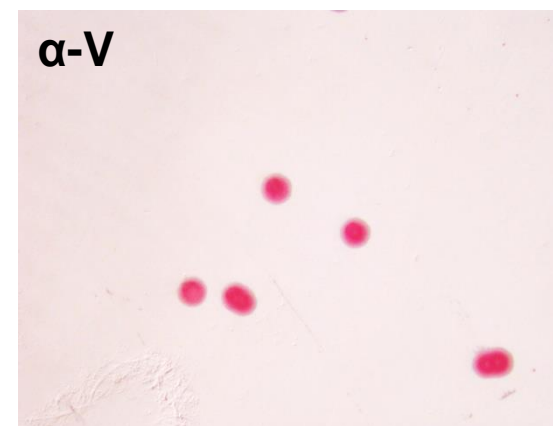

Supplement: S1 Fig — Colonies were positive only when immunostained with VpmaW-specific antisera (α-W) and were negative with all the other five Vpma specific pAbs (α-U, α-Y, α-Z, α-X and α-V) and were counterstained in pink using the non-specific protein dye (Ponceau S). (PDF) [file ppat.1006656.s001.pdf]

$\alpha$ -Y

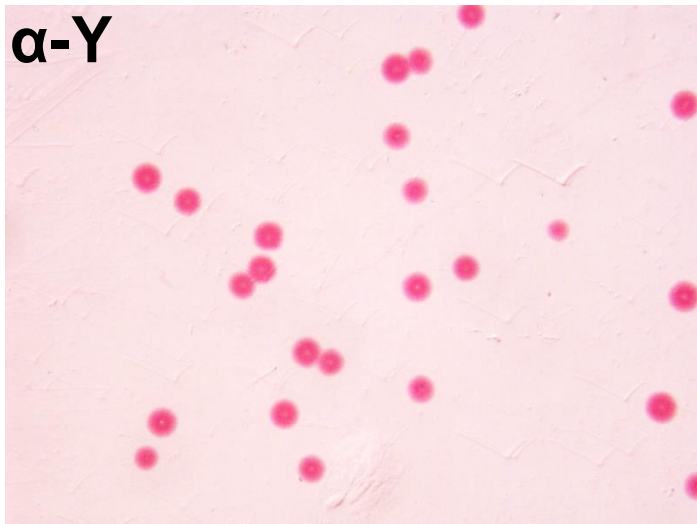

$\alpha$ -U

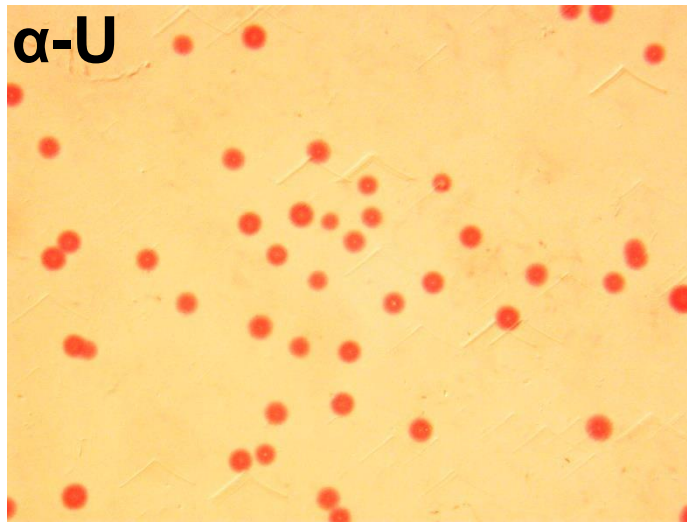

Supplement: S2 Fig — All colonies were negative for immunostaining using VpmaY- and VpmaU-specific α-Y and α-U antisera, respectively, and appeared pink with the non-specific Ponceau S counterstaining. (PDF) [file ppat.1006656.s002.pdf]

**$\alpha$ -W**

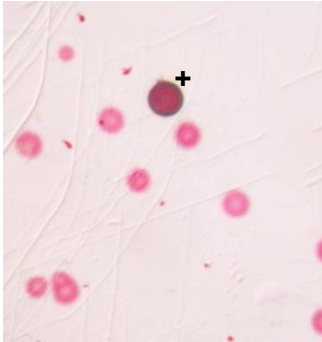

**$\alpha$ -U**

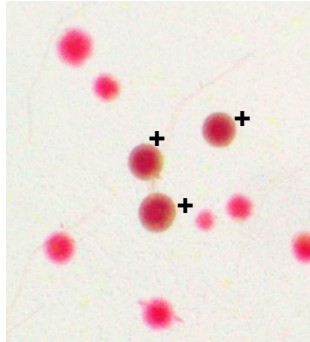

**$\alpha$ -Y**

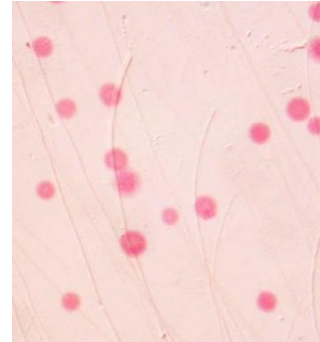

**$\alpha$ -Z**

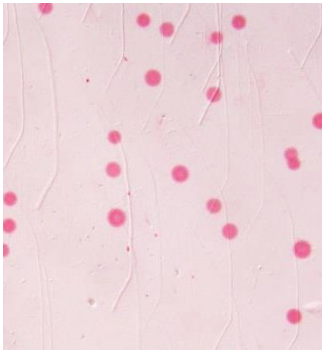

**$\alpha$ -X**

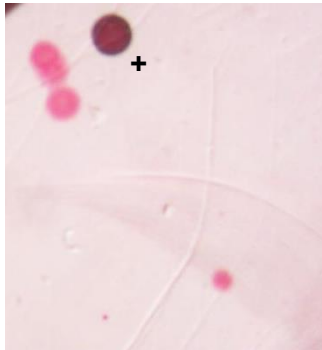

**$\alpha$ -V**

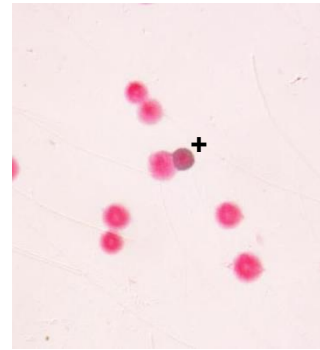

Supplement: S3 Fig — Many colonies were positive when immunostained with α-U pAb and were completely negative with α-Y and α-Z, whereas singular positive colonies were observed with α-W, α-X and α-V antisera. Negative colonies were counterstained in pink using the non-specific protein dye (Ponceau S). (PDF) [file ppat.1006656.s003.pdf]

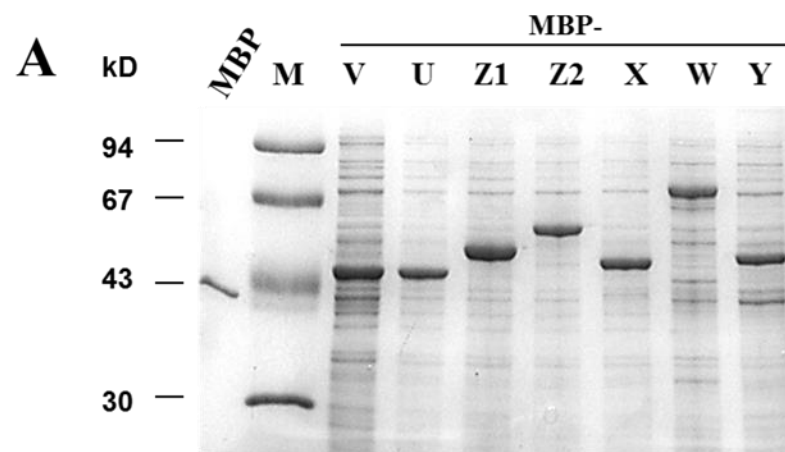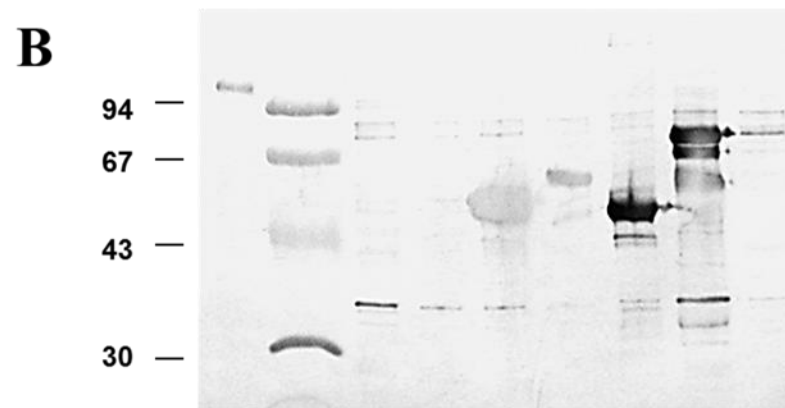

Supplement: S4 Fig — (A) Expression of MBP (Maltose Binding Protein)-Vpma fusion proteins [13] as observed on Coomassie blue-stained reducing SDS-polyacrylamide gel. Z1 and Z2 correspond to two different (regions) VpmaZ-MBP fusion proteins. (B) Immunostaining with polyclonal anti-M. agalactiae serum PAL-97 obtained from a naturally infected sheep [37]. (PDF) [file ppat.1006656.s004.pdf]
