# Supplementary material for: Vpma phase variation is important for survival and persistence of Mycoplasma agalactiae in the immunocompetent host
Source: PLoS Pathog. 2017 Sep 28;13(9):e1006656. doi: 10.1371/journal.ppat.1006656 (PMC5634654; doi:10.1371/journal.ppat.1006656)
Supplement: S1 Table — (DOCX) [file ppat.1006656.s005.docx]

**S1 Table. Oligonucleotide sequences used in this study**

| **Name** | **Sequence (5’ to 3’)^a^** | **Source/Reference** |
| --- | --- | --- |
| 1618P1 | GCCCCATAATTAGAGCCC | This study |
| P2BPLMY | GGTGGTAGCACATCAACTG | This study |
| P2PLMU | TTTGAAGATGTGTTGGATG | This study |
| P6PLMU | CTCCAGCAGAAGGCAGTC | This study |
| P6PLMY | AGCGTTTGCATTGGCTAC | This study |
| P7PLMU | GTTACATTTACTGGAACTG | This study |
| P7PLMY | TTTTGTTGCCTAGTTTCTG | This study |
| P8PLMU | TAACGTCAGCAATGTCTG | This study |
| P8PLMY | TTATAGCCATTAAAGAAAGG | This study |
| P9PLMU | AGGCCTCATAATTATGAAG | This study |
| P9PLMY | AGTTTAGATTGACCTTTGG | This study |
| RecendET28 | GACGAGAAGCTTACTATTAAGCATTATTTTTC | [43] |
| T3ISLrev | AGAGCAGAATTCAATTAACCCTCACTACTAAAG | [43] |
| Prlocol1 | CAAGTTATCAAGTTGCATGTAG | This study |
| XerS | GCTAGGTCTAGATAGAGTGATATACGACAC | [13] |
| XerR | TACTGTGGTACCTAGACTATTGATGCTTAC | [13] |
| U2F | CGCGGATCCGATAAAGAAGATAAGACAGGTG | [13] |
| Urev1 | TCAACCTTAGATAAATCACCTAAC | This study |
| Urevvw4 | GTTCTATCATGTCACTAGTTTG | This study |
| Yrevpr5 | ATTAGTAGAAGATTGTAGCG | This study |
| Yrevv8 | TTGTGCTTGCCTGTTACC | This study |
| Z1F | CGCGGATCCCAAACAGATTCAACTCCGTCAAC | [20] |
| Z1R | AAACTGCAGTTATTCGTATTTAGGTAATAGTCTTC | [20] |
| Zend1 | AAGACTATTACCTAAATACG | This study |
| Vendrv | TAAATTTGAAGTATAAGTGA | This study |
| Wstfw | TGATTTCTTTTCGTTTGTTG | This study |
| WDIGrv | AGAAGCATTTAAAGTGAATGAAACC | This study |
| WDIGfw | TGATTTCTTTTCGTTTGTTG | This study |
|  |  |  |
| X1F | CGCGGATCCAAAGTAATGAAGGTCAATTACC | [20] |
| X1R | AAACTGCAGGCTTAAGGATTTTTTAAAATGATG | [20] |
| PrSq1 | AGCAAGGCAAAATTCAGAATATCTC | This study |
| PrSq2 | AACTAAAACATTCTGTAATCTTAG | This study |
| Y3F | CCGGAATTCAATGCAAACGCTGCAGAAAATG | [13] |
| Y3R | GCTCTAGATTAAGTAAATGTAACTGTAACTTCACC | [13] |
| TetMEnd | ACGCATAGTAGACCACCTC | This study |
